# Redox-Stable and Multicolor Electrochromic Polyamides with Four Triarylamine Cores in the Repeating Unit

**DOI:** 10.3390/polym16121644

**Published:** 2024-06-11

**Authors:** Yaw-Terng Chern, Chien-Cheng Yen, Jia-Mao Wang, I-Shan Lu, Bo-Wei Huang, Sheng-Huei Hsiao

**Affiliations:** 1 Department of Chemical Engineering, National Taiwan University of Science and Technology, Taipei 106335, Taiwan; m9806027@mail.ntust.edu.tw (C.-C.Y.); m10006039@mail.ntust.edu.tw (J.-M.W.); hunhan312613@gmail.com (I.-S.L.); 2 Department of Chemical Engineering and Biotechnology, National Taipei University of Technology, Taipei 10608, Taiwan; s0979121791@gmail.com

**Keywords:** electrochromic polymers, long-term switching stability, aromatic polyamides, triphenylamine

## Abstract

Two new triarylamine-based diamine monomers, namely, *N*,*N*′-bis(4-methoxyphenyl)-*N*,*N*′-bis(4-(4-aminophenyl-4′-methoxyphenylamino)phenyl)-*p*-phenylenediamine (**3**) and *N*,*N*′-bis(4-methoxyphenyl)-*N*,*N*′-bis(4-((4-aminophenyl-1-naphthyl)amino)phenyl)-*p*-phenylenediamine (**7**), were successfully synthesized and led to two series of electroactive polyamides by polycondensation reactions with common aromatic dicarboxylic acids. The polymers demonstrated multicolored electrochromism, high optical contrast, and remarkable enhancements in redox and electrochromic stability. Compared to other triarylamine-based polymers, the studied polyamides exhibited enhanced electrochromic stability (only 3~6% decay of its coloration efficiency at 445 nm after 14,000 switching cycles) at the first oxidation stage. The polyamides also showed strong absorption in the near-infrared region upon oxidation. Polymers with multicolored electrochromism and high redox stability can be developed by incorporation of four triarylamine cores in each repeat unit and electron-donating methoxy groups on the active sites of the triphenylamine units.

## 1. Introduction

Electrochromic materials have attracted great attention due to their reversible optical change properties [1,2]. It has been demonstrated that organic conjugated polymers and viologens used as electrochromic materials have more advantages than inorganic compounds, including high CE, fast switching speed, easy of large-area processing, and easy color tuning through chemical modification [3,4,5,6,7,8,9]. Therefore, they have promising applications in various fields, such as smart windows, memory devices, rear view mirrors, and information displays. In addition, TPA-based polymers have been continuously and extensively developed as a new and attractive family of electrochromic materials because of their colorless neutral state and interesting electrochromic behavior, such as high coloration efficiency, rapid switching speed, ease of processing, and easy color tuning through chemical modification [10,11,12,13,14,15,16,17,18,19,20]. Variable infrared optical property technology has attracted increasing interest due to the promising applications in commercial thermal control and environmentally adaptable military camouflage [21]. In recent years, researchers have developed into near-infrared (NIR) range and explored the potential application of electrochromic in this range [12,14,22,23,24]. Smart windows based on NIR electrochromic materials could regulate the intensity of the transmitted NIR radiation, reduce air conditioning costs, and provide environmental benefits [25].

Robin and Days classified mixed-valence compounds containing two or more oxidation centers into three categories. The *N*,*N*,*N*′,*N*′-tetraphenyl-*p*-phenylenediamine (TPPA) cation radical has been reported as a symmetrical delocalized class III structure with a strong electronic coupling (the electron is delocalized over the two redox centers) [26]. Nelson and Adam et al. demonstrated that the unsubstituted TPA moieties undergo dimerization to tetraphenylbenzidine after formation of cation radical [27,28]. When the phenyl groups were incorporated by electron-donating substituents at the *para*-position of TPA, the dimerization was greatly prevented by affording stable cationic radicals [29,30]. Although the influence of the substituents on electrochromic stability of materials have been reported, the structure/electrochromic stability relationships for TPA-based polymers are still poorly understood and mostly unexplored.

In the development of electrochromic materials, several critical factors are considered, including electrochromic stability, high CE, rapid switching speed, and a significant change in optical transmittance during operation. Among the above characteristics, the electrochromic stability of the polymer is a crucially important feature for practical use of materials for any purposes [31]. TPA-based electrochromic materials have been developed for decades. However, most of them usually have limited electrochromic stability. For example, the majority of the TPA-based polymers reveal that their electroactivity was retained at 90% after only 2000 switching cycles [12,13,14,15,16,17,18,19]. However, TPA-based polymers derived from only two specific diamines have demonstrated significantly better electrochromic stability with their electroactivity, decaying less than 10% even after 10,000 switching cycles [12,14]. As a result, achieving electrochromic stability in TPA-based polymers remains a challenging task, and future research is required to address this issue effectively.

**Figure 1:**
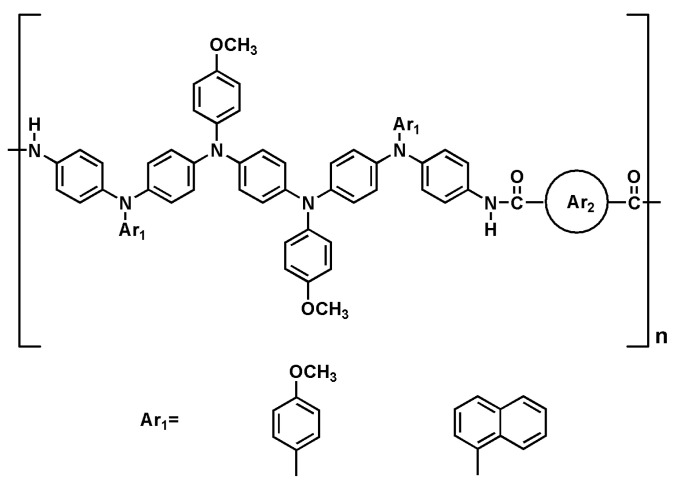

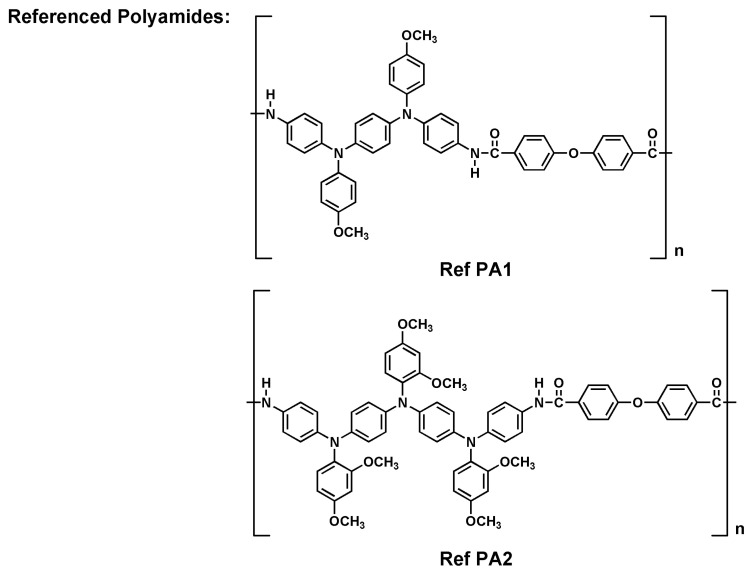
Structures of the polyamides studied in this work and the referenced ones.

The objective of this research is to develop organosoluble PAs with high electrochromic stability, fast switching speed, high CE, and multiple electrochromic changes. The electrochromic stability of materials is a crucial factor for their practical applicability. To enhance the stability of electrochromic PA materials, this study provides strategies, including the introduction of four electroactive triarylamino cores in the repeating unit. Multicolored electrochromic materials, which allow for multicolor tunability, have attracted considerable research attention for their potential applications in displays. This work aimed to obtain high electrochromic stability and multiple colors within the same electrochromic material. The colored state can be modulated effectively by incorporating different substituents to adjust conjugation length. Therefore, we focused on specifically designed TPA-based PAs with four electroactive triarylamine centers containing 4-methoxyphenyl or 1-naphthyl groups in the repeating unit. The influence of the substituents and number of electroactive nitrogen centers on electrochromic stability of the PAs was also investigated. Physical properties such as the thermal stability, electrochemical stability, and spectroelectrochemical stability were investigated. Some properties of the present PAs will be compared with previous studies reporting on the TPA-based referenced polymers **Ref PA1** [11] and **Ref PA2** [32] (Figure 1).

## 2. Materials and Methods

*N*-Methyl-2-pyrrolidone (NMP) and dimethyl sulfoxide (DMSO) were dried by CaH_2_, followed by distillation under reduced pressure. Calcium chloride was dried under a vacuum at 200 °C for 10 h. Dry 4 Å molecular sieves are added to pyridine and triphenyl phosphite (TPP) for water removal. Tetrabutylammonium perchlorate (TBAP) was recrystallized by ethyl acetate and then dried in vacuo prior to use. *N*,*N*′-Bis(4-(4-nitrophenylamino)phenyl)-*p*-phenylenediamine (**1**), 9,9-bis(4-(4-carboxyphenoxy)phenyl)fluorine (**8a**), 1,1-bis(4-carboxyphenoxyphenyl)-1-phenyl-2,2,2-trifluoro-ethane (**8b**), 2,2′-bis(trifluoromethyl)-4,4′-dicarboxybiphenyl (**8c**), and referenced polyamides **Ref PA1** and **PA2** were synthesized according to reported procedures [12,32,33,34,35,36]. All other reagents were used as received from commercial sources.

### 2.1. Synthesis N,N′-Bis(4-methoxyphenyl)-N,N′-bis[4-(4-methoxyphenyl-4′-nitrophenylamino)phenyl]-p-phenylenediamine (***2***)

A 150 mL three-neck flask was charged with 5.0 g (9.4 mmol) of *N*,*N*′-bis(4-(4-nitrophenylamino)phenyl)-*p*-phenylenediamine (**1**), 11 g (47 mmol) of 4-iodoanisole, 3 g of copper powder, 6.5 g of K_2_CO_3_, 1.0 g of 18-crown-6-ether, and 15 mL of *o*-dichlorobenzene. The mixture was heated under reflux in nitrogen atmosphere for 24 h. The mixture was then filtered to remove the copper powder, and the filtrate was allowed to cool, resulting in the precipitation of the reddish-brown crude product. The crude product was recrystallized from *N*,*N*-dimethylformamide (DMF) and methanol to afford 3.2 g (36% in yield) of reddish-brown powder: mp 296–298 °C. IR (KBr): 3038 (sp^2^C–H str.), 2949, 2835 (sp^3^C–H str.), 1589 (sym. –NO_2_ str.), 1311 cm^−1^ (asym. –NO_2_ str.). Anal. Calcd for C_58_H_48_N_6_O_8_ (wt%): C, 72.8; H, 5.02; N, 8.79. Found: C, 72.4; H, 5.01; N, 8.74. ^1^H NMR (CDCl_3_, δ, ppm): 3.81, 3.83 (s, 12H), 6.79 (d, *J* = 9.3 Hz, 4H), 6.87 (d, *J* = 8.9 Hz, 4H), 6.92 (d, *J* = 8.9 Hz, 4H), 6.95–7.05 (m, 12H), 7.11 (d, *J* = 8.9 Hz, 4H), 7.15 (d, *J* = 8.9 Hz, 4H), 8.00 (d, *J* = 9.3 Hz, 4H). ^13^C NMR (CDCl_3_, δ, ppm): 55.49, 55.52, 114.90, 115.25, 116.01, 122.21, 124.77, 125.56, 127.17, 127.33, 128.34, 138.19, 138.24, 139.07, 140.16, 142.59, 146.14, 154.02, 156.44, 157.79.

### 2.2. Synthesis of N,N′-Bis(4-methoxyphenyl)-N,N′-bis(4-(4-aminophenyl-4′-methoxyphenylamino)phenyl)-p-phenylenediamine (***3***)

In a 150 mL three-neck flask, 3.2 g (3.35 mmol) of dinitro compound **2**, 0.25 g of Pd/C, and 40 mL of NMP were added; the mixture was stirred under hydrogen atmosphere at room temperature until the theoretical amount of hydrogen was consumed. The time to reach this stage is about 72 h. The mixture was then filtered to remove Pd/C, and the filtrate was allowed to cool, resulting in the precipitation of the light green crude product. The crude product was recrystallized from DMF and methanol to afford 2.1 g (70% in yield) of light green powder: mp 271–274 °C. IR (KBr): 3448, 3364 (–NH_2_ str.), 3038 (sp^2^C–H str.), 2949, 2835 cm^−1^ (sp^3^C–H str.). Anal. Calcd for C_58_H_52_N_6_O_4_ (wt%): C, 77.7; H, 5.80; N, 9.38. Found: C, 77.2; H, 5.76; N, 9.33. ^1^H NMR (600 MHz, DMSO-*d*_6_, δ, ppm): 3.70 (s, 12H), 4.96 (s, 4H), 6.52 (d, *J* = 8.8 Hz, 4H), 6.67 (d, *J* = 8.8 Hz, 4H), 6.77–6.68 (m, 20H), 6.91–6.96 (m, 8H). ^1^H NMR (500 MHz, pyridine-*d*_5_, δ, ppm): 3.68 (s, 6H), 3.69 (s, 6H), 6.95 (m, 12H), 7.14 (m, 16H), 7.22 (d, 4H), 7.26 (d, *J* = 8.8 Hz, 4H). ^13^C NMR (125 MHz, pyridine-*d*_5_, δ, ppm): 55.77, 115.47, 115.61, 116.34, 122.46, 124.21, 125.32, 125.95, 126.67, 128.09, 138.42, 141.98, 142.09, 142.59, 143.55, 145.06, 146.37, 156.00, 156.43.

### 2.3. Synthesis of N,N′-Bis(4-methoxyphenyl)-N,N′-bis[4-(4-nitrophenylaminopheny]-p-phenylenediamine (***5***)

In a 150 mL three-neck flask, 5 g (9.9 mmol) of *N*,*N*′-bis(4-aminophenyl)-*N*,*N*′-bis(4-methoxyphenyl)-*p*-phenylenediamine (4), 3.51 g (24.9 mmol) of 4-fluoronitrobenzene, 4.23 g of triethylamine (TEA), and 20 mL of DMSO were added. The mixture was heated at 120 °C for 72 h. The mixture was allowed to cool, resulting in the precipitation of the brown crude product. The crude product was recrystallized from DMF to afford 5.8 g (79% in yield) of brown powder: mp 242–244 °C. IR (KBr): 3354 (N–H str.), 3037 (sp^2^C–H str.), 2902, 2833 (sp^3^C–H str.), 1594 (sym. –NO_2_ str.), 1302 cm^−1^ (asym. –NO_2_ str.). ^1^H NMR (500 MHz, DMSO-*d*_6_, δ, ppm): 3.73 (s, 6H), 6.94 (m, 16H), 7.06 (d, *J* = 7.5 Hz, 4H), 7.14 (d, *J* = 7.5 Hz, 4H), 8.06 (d, *J* = 7.5 Hz, 4H), 9.18 (s, 2H). ^13^C NMR (125 MHz, DMSO-*d*_6_, δ, ppm): 55.16, 112.70, 114.96, 122.60, 123.80, 126.14, 126.62, 133.51, 137.26, 139.92, 142.05, 143.86, 151.28, 155.70, 162.21.

### 2.4. Synthesis of N,N′-Bis(4-methoxyphenyl)-N,N′-bis[4-((1-naphthyl-4-nitrophenyl)amino)phenyl]-p-phenylenediamine (***6***)

A 150 mL three-neck flask was charged with 5.8 g (7.8 mmol) of compound 5, 5.9 g (23.4 mmol) of 4-iodoanisole, 1.2 g of copper powder, 2.7 g of K_2_CO_3_, 3.5 g of 18-crown-6-ether, and 17 mL of *o*-dichlorobenzene. The mixture was heated to reflux under nitrogen atmosphere for 24 h. The mixture was then filtered to remove the copper powder, and the filtrate was allowed to cool, resulting in the precipitation of the orange crude product. The crude product was recrystallized from DMF to afford 6.2 g (80% in yield) of orange powder: mp 250–252 °C. IR (KBr): 3036 (sp^2^C–H str.), 2904, 2831 (sp^3^C–H str.), 1591 (sym. –NO_2_ str.), 1305 cm^−1^ (asym. –NO_2_ str.). ^1^H NMR (500 MHz, DMSO-*d*_6_, δ, ppm): 3.68 (s, 6H), 6.60 (d, *J* = 7.0 Hz, 4H), 6.85 (d, *J* = 7.0 Hz, 4H), 6.91 (m, 8H), 7.04 (d, *J* = 6.5 Hz, 4H), 7.24 (d, *J* = 7.0 Hz, 4H), 7.51 (t, 2H), 7.57 (m, 4H), 7.62 (t, 2H), 7.86 (t, 2H), 8.00 (m, 6H), 8.05 (d, *J* = 8.5 Hz, 2H). ^13^C NMR (125 MHz, DMSO-*d*_6_, δ, ppm): 55.14, 114.65, 115.04, 121.33, 122.74, 124.75, 125.68, 126.61, 126.70, 126.72, 126.91,127.28, 127.30, 128.11, 128.73, 129.81, 134.75, 137.17, 138.11, 139.26, 140.41, 142.07, 145.88, 154.00, 156.14.

### 2.5. Synthesis of N,N′-Bis(4-methoxyphenyl)-N,N′-bis(4-((4-aminophenyl-1-naphthyl)amino)phenyl)-p-phenylenediamine (***7***)

In a 150 mL three-neck flask, 5.8 g (3.35 mmol) of compound 6, 0.46 g of Pd/C, and 18 mL of NMP were added, the mixture was stirred under hydrogen atmosphere at room temperature until the theoretical amount of hydrogen was consumed. The time to reach this stage is about 120 h. The mixture was then filtered to remove the Pd/C, and the filtrate was allowed to cool, resulting in the precipitation of the light green crude product. The crude product was recrystallized from DMF to afford 3.9 g (71% in yield) of light green powder: mp 188–191 °C. IR (KBr): 3445, 3367 cm^−1^ (–NH_2_ str.). ^1^H NMR (500 MHz, DMSO-*d*_6_, δ, ppm): 3.74 (s, 6H), 4.91 (s, 4H), 6.51 (m, 8H), 6.72 (m, 8H), 6.81 (m, 8H), 6.90 (d, *J* = 7.0 Hz, 4H), 7.26 (d, *J* = 7.0 Hz, 2H), 7.37 (t, 2H), 7.46 (m, 4H), 7.74 (t, 2H), 7.93 (m, 4H). ^13^C NMR (125 MHz, DMSO-*d*_6_, δ, ppm): 55.13, 114.71, 114.78, 119.29, 122.68, 123.86, 124.49, 125.21, 125.41, 125.74, 125.83, 125.98, 126.02, 126.45, 128.32, 130.41, 134.82, 136.55, 139.68, 140.69, 142.00, 143.82, 145.04, 145.10, 154.89.

### 2.6. Polymer Synthesis

Using the synthesis of PA **10d** as an example: In a 100 mL three-neck flask, 0.936 g (1 mmol) of diamine monomer **7**, 0.259 g (1 mmol) of 4,4′-dicarboxydiphenyl ether (**8d**), 3.0 mL of NMP, 1.2 mL of TPP, 0.4 mL of pyridine, and 0.15 g of CaCl_2_ were added. The reaction was carried out at 120 °C under nitrogen atmosphere for 6 h. The obtained polymer solution was poured into 40 mL of stirred methanol, resulting in the formation of a stringy, fiber-like precipitate. The precipitate was washed several times with hot water, and then vacuum-dried. The obtained PA **10d** was dissolved in *N*,*N*-dimethylacetamide (DMAc) to prepare an 8 wt% solution. The solution was again precipitated in methanol for purification. The inherent viscosity of the purified **7d** was measured to be 0.47 dL/g using NMP as the solvent. IR (KBr): 1655 (C=O str.), 3316 cm^−1^ (amide N–H str.). ^1^H NMR (pyridine-*d*_5_, δ, ppm): 3.76 (12H), 6.85–6.99 (24H), 7.15 (4H), 7.30–7.59 (12H), 7.81–7.98 (10H), 10.09 (2H, CONH).

### 2.7. Measurements

A Bio-Rad 3240-SPC FTIR spectrophotometer was used to record IR spectra (KBr pellets). In a typical experiment, an average of 20 scans per sample was made. ^1^H and ^13^C NMR spectra were recorded on a Bruker AV 500 Fourier transform nuclear magnetic resonance spectrometers using tetramethylsilane (TMS) as the internal standard. A PerkinElmer 240C elemental analyzer was used for elemental analysis. The melting points were obtained by a standard capillary melting point apparatus. Inherent viscosities of all polymers were determined at 0.5 g/dL using an Ubbelohde viscometer. Qualitative solubility was determined using 0.01 g of polymer in 1.0 mL of solvent. A TA instruments DSC 2010 differential scanning calorimeter and a PerkinElmer Diamond S II thermogravimetric analyzer were employed to study the transition data and thermal decomposition temperature of all the polymers. A differential scanning calorimeter (DSC) was run under a nitrogen stream at a flow rate 30 cm^3^/min and a heating rate of 20 °C/min. Thermogravimetric analysis (TGA) was determined under a nitrogen flow of 50 cm^3^/min. Thermal mechanical analysis (TMA) was performed on a TA Instruments 2940 thermomechanical analyzer at a scan rate of 5 °C/min. Electrochemistry was performed using a CH instruments 611C electrochemical analyzer. Cyclic voltammetry (CV) measurements were conducted using polymer film/indium-tin (ITO) as a working electrode, a Ag/AgCl, KCl (sat) as a reference electrode, and a platinum wire as an auxiliary electrode. Spectroelectrochemical measurements were carried in a cell built from a 1 cm UV-visible cuvette using a UV Perkin Elmer for Lambda 900 spectrophotometer. The polymer film/ITO was used as a working electrode, a platinum as a counter electrode, and Ag/AgCl as a reference electrode.

## 3. Results and Discussion

### 3.1. Monomer Synthesis

**Figure 2:**
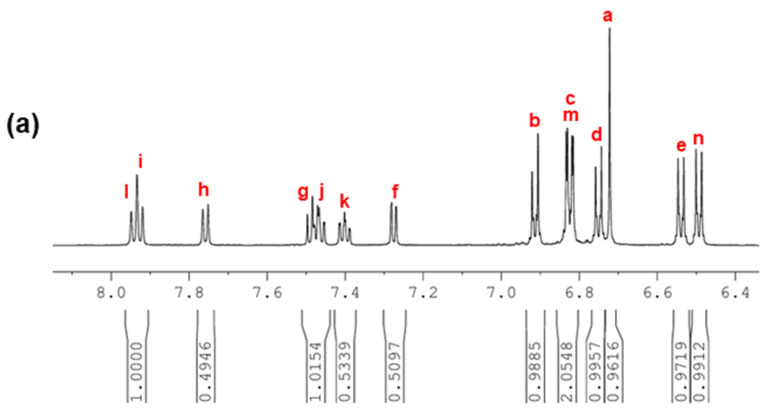

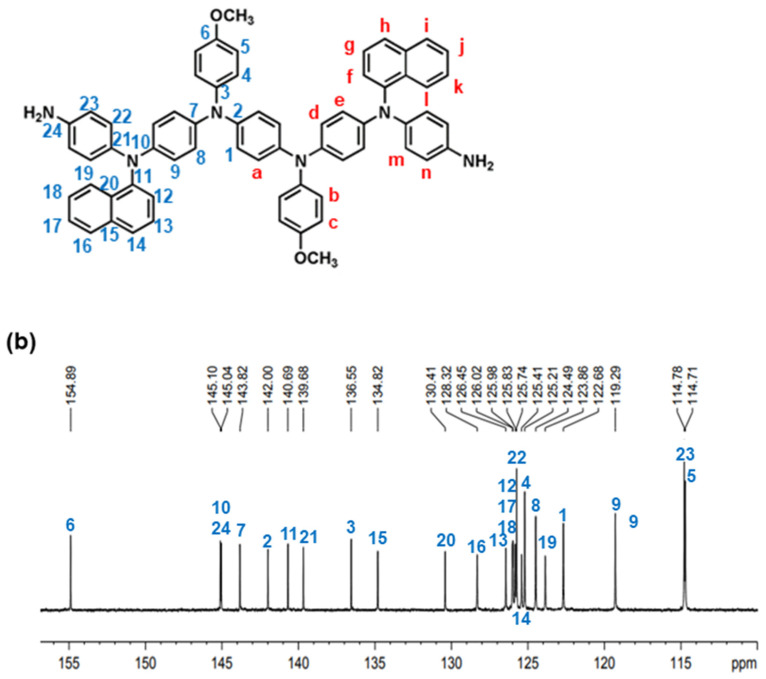
(**a**) ^1^H and (**b**) ^13^C NMR spectra of diamine monomer **7**. Signals of aryl amino and methoxy protons at 4.91 and 3.67 ppm, respectively, and methoxy carbons at 55.13 ppm are found in its ^1^H and ^13^C NMR spectra.

**Scheme 1:**
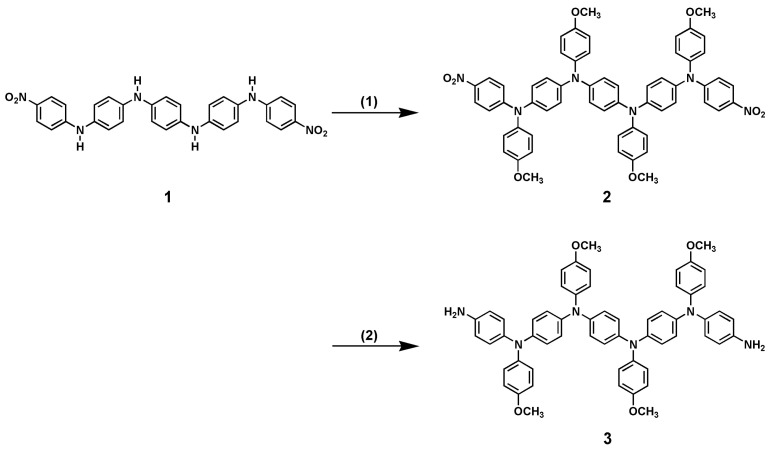
Synthetic route to diamine monomer **3**: (1) 4-iodoanisole, Cu, K_2_CO_3_, o-dichlorobenzene, 18-crown-6 ether; (2) H_2_, Pd/C, NMP.

**Scheme 2:**
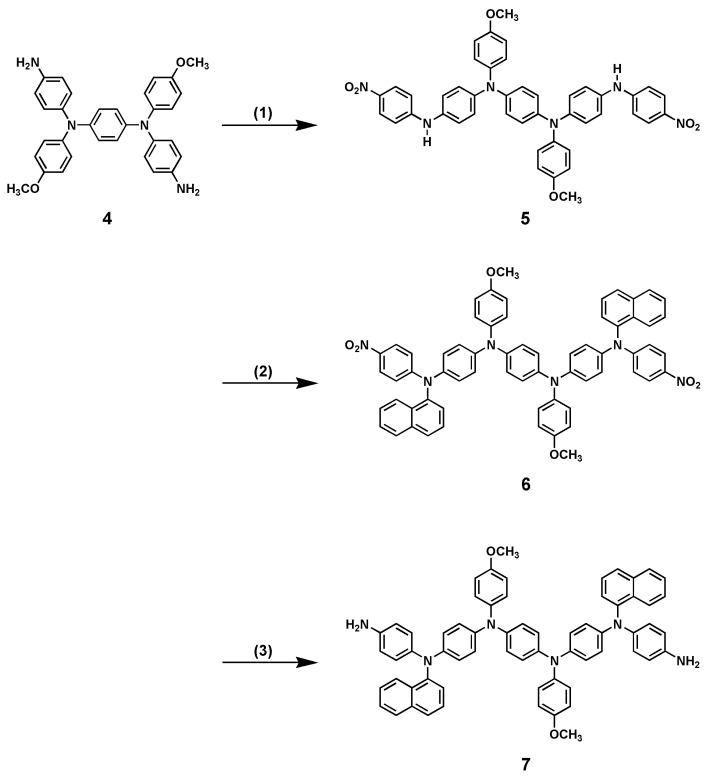
Synthetic route to diamine monomer **7**: (1) *p*-fluoronitrobenzene, triethylamine, DMSO; (2) 1-iodonaphthalene, Cu, K_2_CO_3_, o-dichlorobenzene, 18-crown-6-ether; (3) H_2_, Pd/C, DMF.

The two new diamine monomers of *N*,*N*′-bis(4-methoxyplenyl)-*N*,*N*′-bis(4-(4-aminophenyl-4′-methoxyphenylamino)phenyl)-*p*-phenylenediamine (**3**) and *N*,*N*′-bis(4-methoxyphenyl)-*N*,*N*′-bis(4-((4-aminophenyl-1-naphthyl)amino)phenyl)-*p*-phenylenediamine (**7**) were synthesized. Two steps were employed to synthesize the new diamine monomer **3** starting from *N*,*N*′-bis(4-(4-nitrophenylamino)phenyl)-*p*-phenylenediamine (**1**) as shown in Scheme 1. The C–N coupling reaction was performed between compound **1** and 4-iodoanisole in the presence of copper to afford compound **2**. Then the dinitro compound **2** was hydrogenated to generate the diamine monomer **3**. Three steps were employed to synthesize the new diamine monomer **7** from *N*,*N*′-bis(4-aminophenyl)-*N*,*N*′-bis(4-methoxyphenyl)-*p*-phenylenediamine (**4**) as shown in Scheme 2. An aromatic fluoro-displacement reaction of 1-fluoro-4-nitrobenzene with compound **4** in DMSO in the presence of triethylamine (TEA) generated compound **5**. The Ullmann C–N coupling reaction was performed between **5** and 4-iodoanisole, in the presence of copper to afford the dinitro compound **6**. Then, **6** was hydrogenated to yield the new diamine monomer **7**. IR and NMR spectroscopic techniques were used to identify the structures of the intermediate compounds (**2**, **5**, and **6**) and the target diamine monomers (**3** and **7**). The FTIR spectra of the synthesized compounds are shown in Supplementary Materials Figures S1 and S2. The compound **6** exhibited two characteristic bands at around 1591 cm^−1^ and 1305 cm^−1^ due to nitro asymmetric and symmetric stretching. After reduction to the diamine monomer (**7**), the characteristic absorption bands of the nitro group disappeared, and the primary amino group showed amino characteristic bands at 3445 cm^−1^ and 3367 cm^−1^ (–NH_2_ stretching). All the synthesized compounds were also characterized by high-resolution NMR spectroscopy. Typical ^1^H and ^13^C NMR spectra of diamine monomer **7** are illustrated in Figure 2, and the spectra agree well with the proposed molecular structure. The aromatic protons on the benzene rings resonated in the range of 6.49~6.91 ppm, and those of naphthyl appeared at a further downfield region of 7.25~7.95 ppm. The signals appearing at 4.91 and 3.67 ppm are peculiar to the amino and methoxy protons, respectively, of the diamine monomer **7**. The ^13^C NMR spectrum revealed 24 resonance peaks ranging from 1114.71 to 154.89 ppm ascribed to aromatic carbons, together with a signal at 55.13 ppm peculiar to methoxy carbons. The assignments of resonance peaks of diamine monomer 7 were assisted by 2D NMR spectra as shown in Figure S9. The NMR spectra of other synthesized compounds are included in Supplementary Materials Figures S3–S9. The IR and NMR spectra confirmed all the compounds reported herein were successfully synthesized.

### 3.2. Polymer Synthesis

**Scheme 3:**
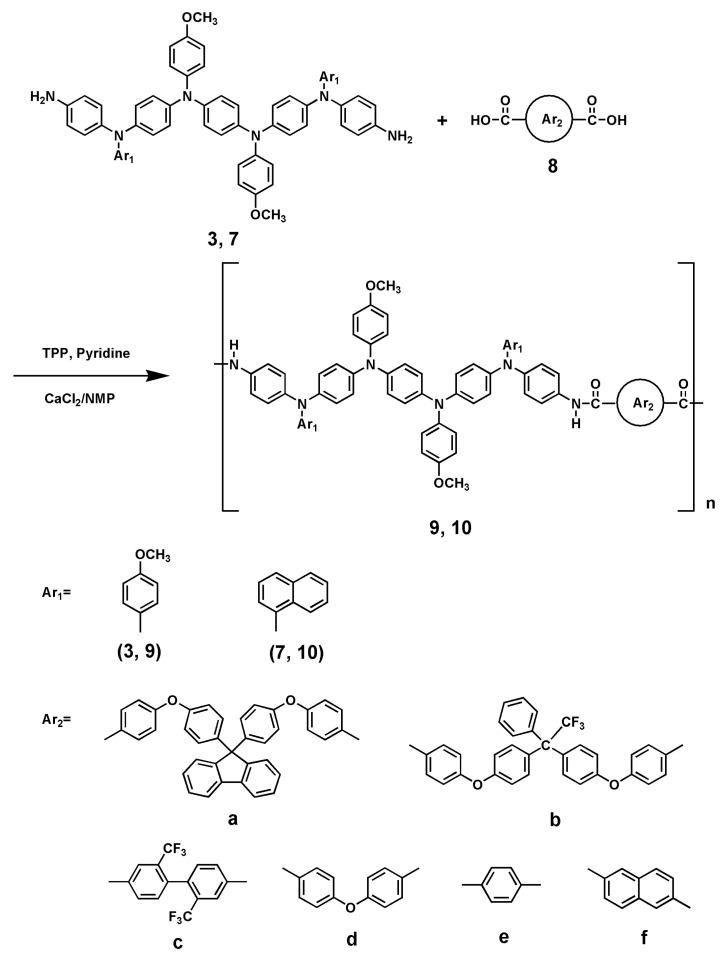
Synthesis of polyamides **9a**–**c** and **10d**–**f**.

According to the phosphorylation method described by Yamazaki et al. [37,38], a series of new aromatic PAs **9a**–**9c** and **10d**–**10f** were synthesized from the diamine monomers **3** and **7**, respectively, with various aromatic dicarboxylic acids (**8a**–**8f**) (Scheme 3). All polymerization reactions proceeded homogeneously and yielded high inherent viscosities. The obtained PAs had inherent viscosities in the range of 0.35–0.65 dL/g (Table S1). All the polymers could afford transparent and tough films via solution casting. At first, we tried to prepare polyamides from diamine 3 and commercially available dicarboxylic acids such as **8d**–**8f**. However, a premature precipitation occurred during the polymerization process, which resulted in low molecular weight products. Thus, dicarboxylic acid monomers **8a**–**8c** were used to react with diamine 3, affording soluble and high molecular weight polyamides **9a**–**9c**.

The formation of PAs was confirmed by IR and ^1^H NMR spectroscopy. A typical IR spectrum of **10e** (see Figure S2) exhibited characteristic absorption bands of the amide group at around 3315 cm^−1^ (N–H str.) and 1656 cm^−1^ (carbonyl str.). Additionally, ^1^H NMR spectra also confirmed the chemical structures of **9c** and **10d** (Figures S10 and S11).

### 3.3. Solubility and Thermal Properties

The solubility of PAs was tested in various solvents, and the solubility data are summarized in Table S1. The PAs **9** and **10** showed good solubility in the tested solvents. All the **10** series PAs were readily soluble in NMP, DMAc, *o*-chlorophenol, and *m*-cresol and also soluble in cyclohexanone on heating at 60 °C. Their good solubility can be attributed to the fact that they contain triphenylamine units and bulky 1-naphthyl side groups, leading to a decrease in the intermolecular force and packing ability of the resulting polymers. The good solubility makes these PAs a potential candidate for practical applications through spin-coating or inkjet printing processes to produce thin films for optoelectronic devices.

Thermal properties of the PAs were evaluated using DSC, TMA, and TGA, and the thermal behavior data are summarized in Table S2. Typical TGA and TMA curves of the representative PA **10d** are shown in Figure S12. All the **9** and **10** series PAs exhibited good thermal stability with no significant weight loss before 400 °C. Their 10% weight-loss temperatures in nitrogen and air were recorded in the range of 421–510 °C and 432–520 °C, respectively. The early loss of weight should be related to the thermal degradation of methoxy and amide groups in the polymer backbone. These PAs showed distinct baseline shifts on their DSC heating traces. Glass transition temperatures (*T*_g_) are defined as the temperature at the midpoint of the baseline shift. These PAs exhibited moderately high *T*_g_ ranging from 204 to 268 °C. The **10** series polymers had higher *T*_g_s as compared to the **9** series ones due to the bulky 1-naphthyl side group. Additionally, the softening temperature (*T*_s_) values of polymer films were determined from the onset temperature of the probe displacement on the TMA trace. The *T*_s_ values were recorded in the range of 195–238 °C for these polymer films by TMA.

### 3.4. Electrochemical Properties

**Figure 3:**
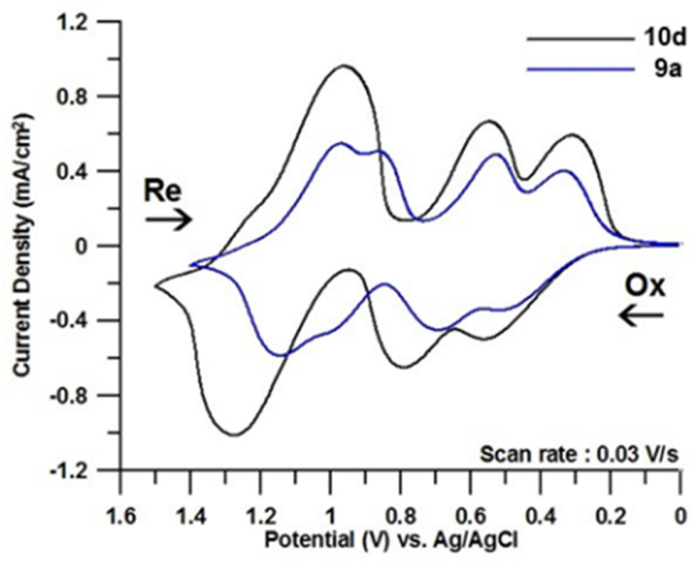
Cyclic voltammograms of PA **9a** and **10d** films at scan rate of 0.03 V/s.

**Figure 4:**
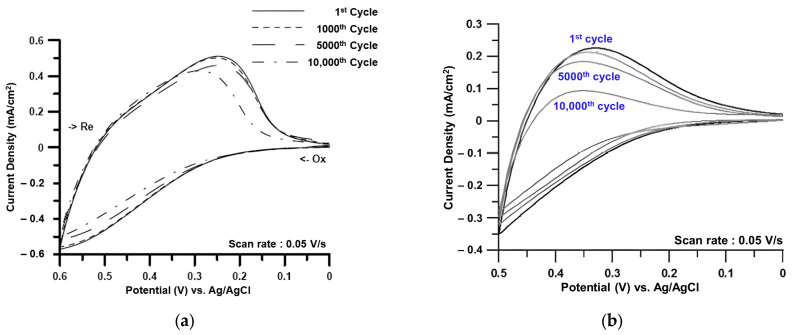
Cyclic voltammograms of PA films at a scan rate of 0.05 V/s: (**a**) **9a** and (**b**) **10d**.

**Figure 5:**
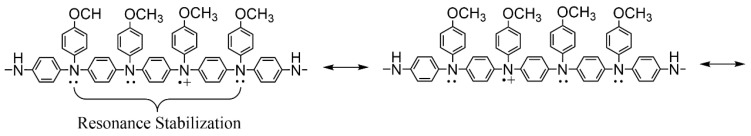
Resonance stabilization forms of the cation radical of the triphenylamine segment in the PA **9a** polymer chain.

The electrochemical behaviors of the PAs were investigated by cyclic voltammetry (CV) measurements, using the polymer film on an ITO as the working electrode and 0.1 M TBAP in anhydrous acetonitrile as the supporting electrolyte. The electrochemical properties of polymers **9** and **10** are summarized in Table 1. Because the electroactivity of the synthesized polymers comes from the triarylamine cores, the electrochemical and electrochromic behaviors in the same series polyamides are very similar. The diacid component seems do not play an important role. Therefore, the electrochemical and electrochromic properties of the representative polyamides **9a** and **10d** are discussed as follows: The typical CV diagrams of PA **9a** exhibit four quasi-reversible redox couples (Figure 3), corresponding to successive removal of electrons from four different nitrogen centers. During the electrochemical oxidation of the PA **9a**, the color of polymer film changed from colorless to yellow–green, dark green, light blue, and navy blue. The first electron removal occurred at the middle nitrogen atom of the four electroactive nitrogen centers because of the highest electron density among the other nitrogen atoms. As shown in Figure 4a, the CV scans of PA **9a** from 0.0 to 0.6 V reveal a highly stable redox process over 10,000 cycles, with only a slight decrease in the peak current. The first redox process exhibits excellent electrochemical stability due to the low redox potential and resonance stabilization of the cationic radical (Figure 5). Additionally, the typical CV diagrams of PA **10d** exhibit three quasi-reversible redox couples (Figure 3). The PA **10d** having four electroactive nitrogen centers revealed only three quasi-reversible redox couples, implying that the third and fourth electrons almost simultaneously removed from the different electroactive nitrogen centers. During the electrochemical oxidation of the PA **10d**, the color of polymer film changed from colorless to yellow–green, grass green, light blue, and navy blue. The first electron removal occurred at the electroactive nitrogen center attaching to the *p*-methoxyphenyl group because of the highest electron density among the other nitrogen atoms. As shown in Figure 4b, the CV scans of **10d** from 0.0 to 0.5 V reveal a highly stable redox process in repetitive 5000 cycles without an obvious decrease in the peak current. **9a** seems to reveal a relatively higher electrochemical stability compared to **10d**. Considering the different chemical structures between **9a** and **10d**, **9a** contains four *p*-methoxyphenyl pendent groups, whereas **10d** contains two *p*-methoxyphenyl and two naphthyl pendent groups. It is well-known that the electron-donating ability of *p*-methoxyphenyl group is stronger than that of naphthyl group. The greater number of *p*-methoxyphenyl groups further help to stabilize the cation radicals of electroactive nitrogen centers for **9a**; thus, **9a** exhibits slightly higher electrochemical stability than **10d**.

The redox potentials of the PAs **9** and **10** and their respective highest occupied molecular orbital (HOMO) and lowest unoccupied molecular orbital (LUMO) (versus vacuum) are calculated and summarized in Table 1. The HOMO level of polymers **9** and **10** could be estimated from the *E*_1/2_ values of their oxidation in CV experiments as 4.78 and 4.74–4.79 eV, respectively, (on the basis of ferrocene/ferrocenium: 4.8 eV below the vacuum level with *E*_1/2_ = 0.44 V). Comparing the homologues **9a** and **Ref PA1** containing TPA moieties, we note that their *E*_onset_ and *E*_1/2_ values at the first stage oxidation decreases as the number of nitrogen on the TPA moieties increases (**9a**: *E*_onset_ = 0.27 V, *E*_1/2_ = 0.42 V; **Ref PA1**: *E*_onset_ = 0.42 V, *E*_1/2_ = 0.54 V [12]). PA **9a** has lower *E*_onset_ and *E*_1/2_ values than those of **Ref PA1**. This is attributed to the fact that the π electron on the phenyl and the unshared electron pair of the nitrogen on TPA form conjugated multiple bonds. Resonance effects are transmitted well between the electroactive TPA units. As a result, the polymers containing more electroactive nitrogen centers exhibit higher electron density of nitrogen on their TPA moieties, due to the electron-donating effect of TPA. The higher electron density results in decreased *E*_onset_ and *E*_1/2_ values.

### 3.5. Spectroelectrochemical Properties

**Figure 6:**
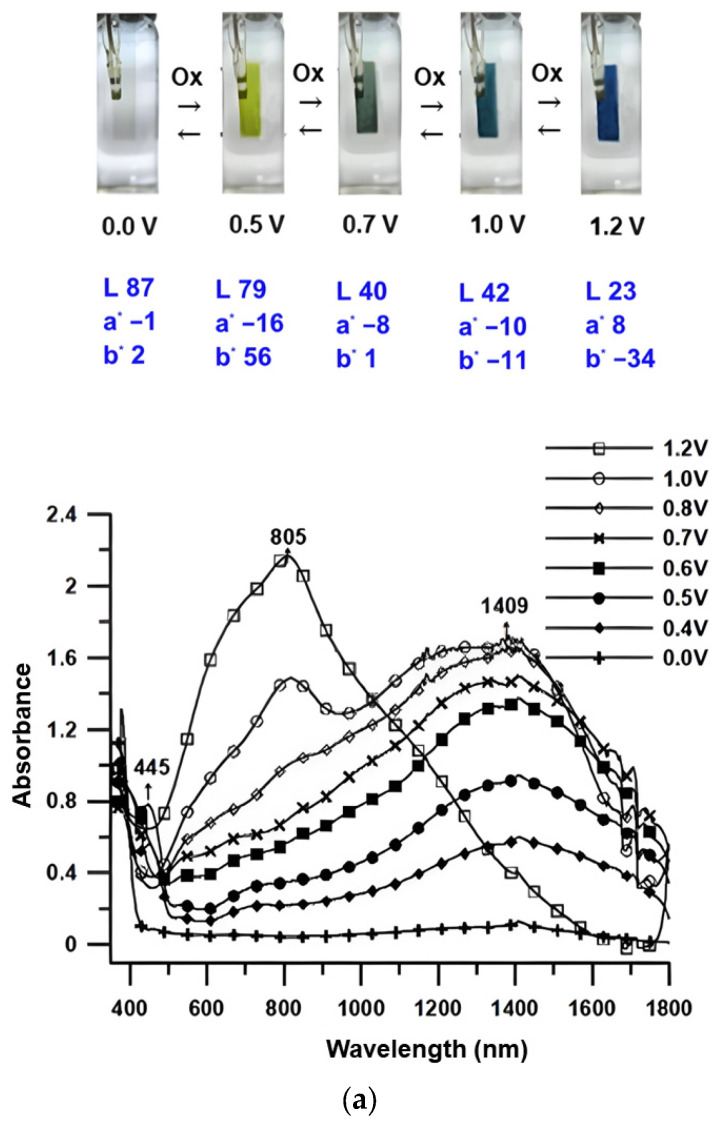

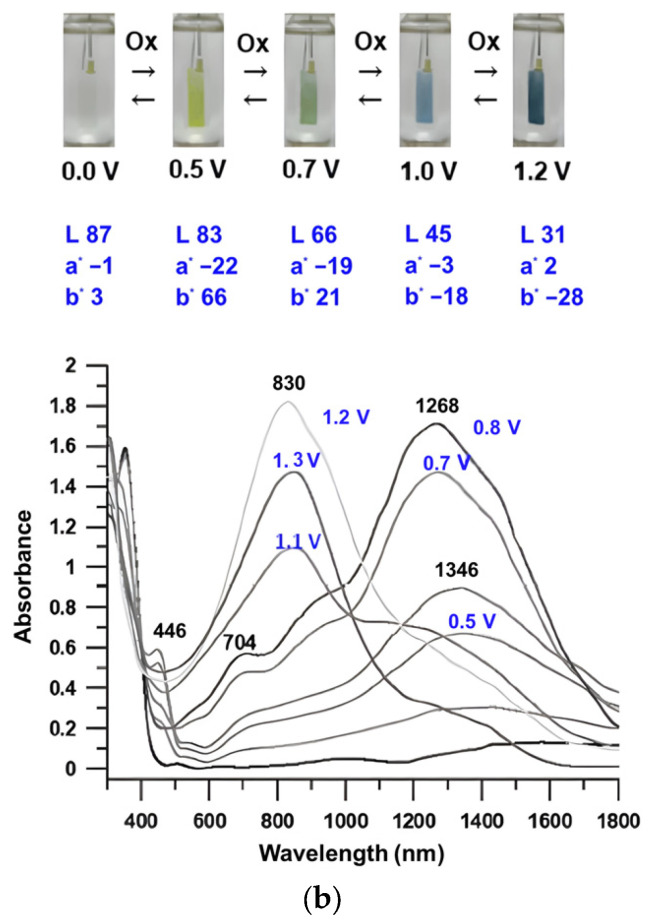
Absorption profile and electrochromic behavior of PAs (**a**) **9a** (thickness ~220 nm) and (**b**) **10d** films (thickness ~150 nm) (in CH_3_CN with 0.1 M TBAP as the supporting electrolyte) at indicated applied voltages.

For the spectroelectrochemical study, the spectra of the polymer film coated on ITO substrate were collected in situ by a UV-vis spectrophotometer under the controlled potentials of an electrochemical analyzer. The typical spectroelectrochemical spectra of PA **9a** are presented in Figure 6a. In the neutral form (0 V), the film exhibited strong absorption at around 350 nm, characteristic of triarylamine, but it was almost transparent in the visible region. Upon oxidation (increasing applied voltage from 0 V to 0.5 V), the intensity of the absorption peak at 350 nm gradually decreased while a new peak at 445 nm and a broad band centered around 1409 nm in the NIR region gradually increased in intensity. The spectral change in the visible-light range is attributed to the formation of a stable monocation radical (**9a^+^**) on the main chain. The electron delocalization over the two nitrogen centers leads to an intervalence charge transfer (IV-CT) absorption in the NIR region. With the potential adjusted to 0.7 V corresponding to the dication **9a^2+^**, the absorption bands (350 nm and 440 nm) decreased gradually, and the absorption band (1409 nm) increased gradually. With the potential adjusted to 1.0 V, the absorption at 1409 nm almost kept the same level, accompanied with a new band centered at around 805 nm. When the potential adjusted to 1.2 V, to achieve a complete oxidation of the four nitrogen centers, the absorption bands at 1409 nm decreased gradually and the absorption band around 805 nm increased in intensity gradually. The disappearance of NIR absorption band can be attributable to the further oxidation of **9a^3+^** species to the formation of **9a^4+^**. As seen in the inset in Figure 6a, the polymer film changed from colorless in the neutral state to yellow–green for the first oxidation state, to dark green for the second oxidation state, to light blue for the third oxidation state, and then to navy blue for the fourth oxidation state. The polymer **9a** shows good contrast in the visible and NIR region with high optical transmittance change (Δ%T) of 66 and 59% at 445 and 1409 nm, respectively, at the first oxidation stage, and 88% at 1409 nm at the second oxidation stage.

As shown in Figure 6b, PA **10d** presented slightly different absorption profiles as compared to PA **9a**. In the neutral form (0 V), the film exhibited strong absorption at around 343 nm, characteristic for triarylamine, but it was almost transparent in visible region. Upon oxidation (increasing applied voltage from 0 V to 0.5 V), the intensity of the absorption peak at 350 nm gradually decreased while a new peak at 446 nm and a broad band centered around 1346 nm in the NIR region gradually increased in intensity. The spectra change in visible-light range is attributed to the formation of a stable monocation radical (**10d^+^**). The electron delocalization over the two nitrogen centers leads to an IV-CT absorption in the NIR region. With the potential adjusted to 0.8 V corresponding to **10d^2+^**, the absorption bands (350 nm and 446 nm) decreased gradually, and the absorption peak gradually shifted to 1268 nm. With the potential adjusted to 1.1 V corresponding to **10d^3+^**, the absorption band at 1268 nm decreased gradually, and a new band centered at around 830 nm emerged. When the potential was adjusted to 1.2 V, to achieve a complete oxidation of the four nitrogen centers, the absorption band at 1268 nm decreased gradually and the absorption band at 830 nm intensified gradually. The disappearance of NIR absorption band can be attributable to the further oxidation of **10d^3+^** species to the formation of **10d^4+^**. As seen in the inset in Figure 6b, the polymer film changed from colorless in the neutral state to yellow–green for the first oxidation stage, to grass green for the second oxidation stage, to light blue for the third oxidation stage, and then to navy blue for the fourth oxidation stage. The polymer **10d** shows good contrast in the visible and NIR region with high optical contrast (Δ%T) of 64 and 78% at 446 and 1346 nm, respectively, at the first oxidation stage, and 92% at 1268 nm at the second oxidation stage.

These triarylamine-based polyamides not only show multicolor electrochromic ability in visible range but also show electrochromic ability in NIR range. Multicolor electrochromism and infrared absorptivity modulation capability make these polymers be attractive materials in IR electrochromic smart windows, optical communication, military camouflage, and thermal control.

### 3.6. Electrochromic Switching Properties

**Figure 7:**
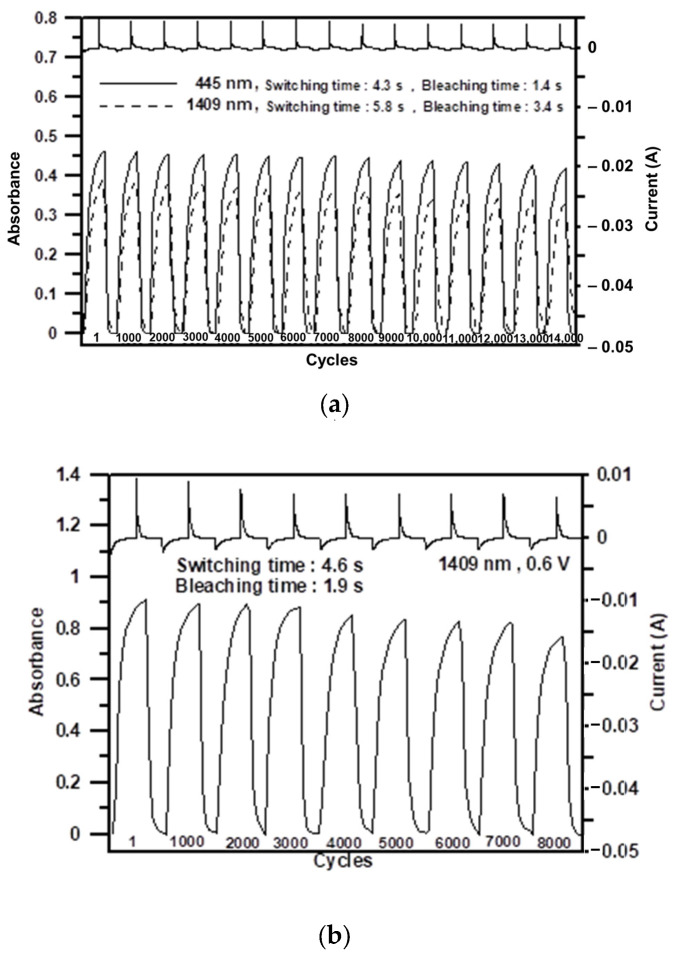
Potential step absorptiometry and current consumption of PA **9a** (in CH_3_CN with 0.1 M TBAP as the supporting electrolyte) by applying a potential step (**a**) 0.0 V ↹ 0.4 V and (**b**) 0.0 V ↹ 0.6 V, and cycle time 20 s.

**Figure 8:**
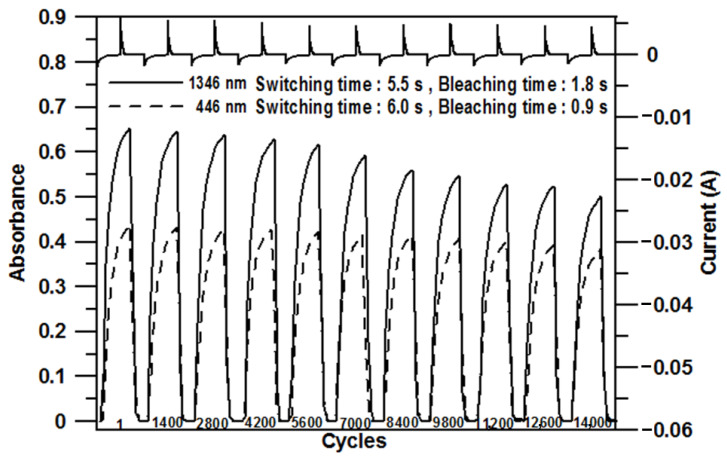
Potential step absorptiometry and current consumption of PA **10d** film (in CH_3_CN with 0.1 M TBAP as the supporting electrolyte) by applying a potential step 0.0 V ↹ 0.5 V, and cycle time 20 s.

**Figure 9:**
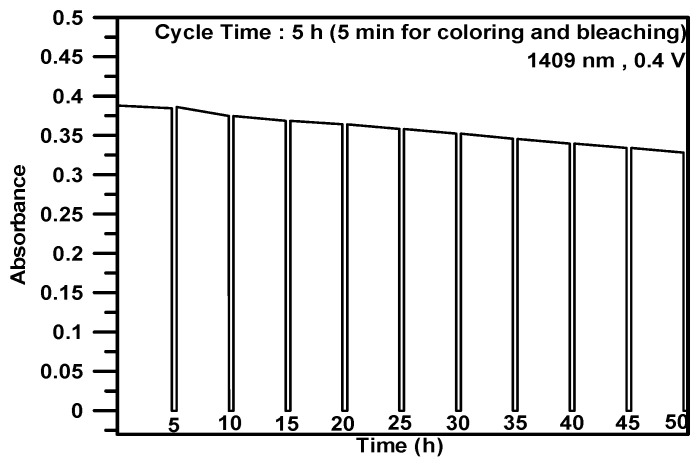
Potential step absorptiometry during the continuous cycling test of PA **9a** (in CH_3_CN with 0.1 M TBAP as the supporting electrolyte) by switching potentials step 0.0 V ↹ 0.4 V with a cycle time of 5 h and 5 min for coloring and bleaching processes, respectively.

The electrochromic switching stability and response time were investigated by monitoring the absorption changes when applying potential steps in the kinetics studies. Switching data for the representative PAs **9a** and **10d** are shown in Figure 7, Figure 8 and Figure S13. The electrochromic coloration efficiency (CE; η = ∆OD/Q) and injected charge after various switching cycles were monitored and are summarized in Table 2, Table 3, Table 4 and Table S3. During the first electrochromic switching between 0.0 and 0.4 V, **9a** exhibited remarkably high switching stability with full reversibility only 3.7% and 4.3% decay of its CE at 445 nm and 1409 nm, respectively, in continuous 14,000 cycles (in Figure 7a). This exceptional stability can be attributed to the stable redox property of **9a** and the good adhesion of the polymer film on the ITO substrate. The detailed discussion regarding the relationship between chemical structure and switching stability is covered in the succeeding section. For the second electrochromic switching between 0.0 and 0.6 V, **9a** also exhibited high switching stability and full reversibility only 8.7% decay of its CE at 1409 nm in continuous 8000 cycles (as depicted in Figure 7b). Furthermore, **9a** exhibited high CE of 272 cm^2^/C and 305 cm^2^/C at 445 nm and 1409 nm, respectively, for the first electrochromic process. Additionally, the response time (coloring/blenching) calculated at 90% of the full optical changes was found to be 5.8/3.4 s and 4.6/1.9 s at 1409 nm for the first and second redox process, respectively. For the first electrochromic switching between 0.0 and 0.5 V, **10d** exhibited remarkably high switching stability with full reversibility only 6.4% and 9.9% decay of its CE at 446 nm and 1346 nm, respectively, in continuous 14,000 cycles (see Figure 8). The exceptional switching stability is attributed to the stable redox property of **10d** and the good adhesion between the polymer film and the ITO substrate. For the second electrochromic switching between 0.0 and 0.7 V, **10d** also exhibited high switching stability and full reversibility only 11% decay of its CE at 1268 nm in continuous 6000 cycles (see Figure S13). **10d** has high CE of 236 cm^2^/C and 381 cm^2^/C at 446 nm and 1346 nm, respectively, for the first electrochromic process. Additionally, the response time (coloring/blenching) calculated at 90% of the full optical changes was found to be 6.0/0.9 s and 5.5/1.8 s at 446 nm and 1409 nm, respectively, for the first redox process. The PAs **9a** and **10d** switched quickly at the first and second stage. Moreover, Figure 9 and Figure S14 illustrate the long-term stability of **9a** and **10d** measured by maintaining 5 h for each coloring process at applied potentials of 0.4 V and 0.5 V, revealing that high stable electrochromic behaviors were observed. For the first oxidation stage, these novel PAs **9a** and **10d** exhibit the highest electrochromic stability to the best of our knowledge compared to all other TPA-based electrochromic polymers. These novel polymers show highly electrochromic stability compared with other electrochromic polymers with similar structures [10,11,12,13].

### 3.7. Relationship between Chemical Structure and Switching Stability

Due to the different number of redox centers, the IV-CT absorption band in the NIR region of materials show changes during the electro-oxidation process. To confirm the relationships between the NIR absorption band and number of the electroactive nitrogen centers, UV-vis-NIR absorbance curves correlated to electrode potentials of PA **9a**, **Ref PA1**, and **Ref PA2** are presented in Figure S15. The λ_max_ and absorption range in the NIR region at the first stage oxidation for these polyamides are summarized in Table 5. Comparing the NIR absorptions of the TPA-based PAs reveals that the λ_max_ and absorption range of these PAs increases as the number of redox centers increases (**9a** > **Ref PA2** > **Ref PA1**). PA **9a** has the longest λ_max_ and widest absorption range among those TPA-based PAs. It has been reported that the TPPA cation radical, being a symmetrical delocalized class III, leads to an IV-CT absorption band in the NIR region where the electron is delocalized over the two redox centers [39,40]. All three polymers of **9a**, **Ref PA1**, and **Ref PA2** are symmetrical delocalized class III. The results confirm that the polymers containing more electroactive nitrogen centers exhibit strong absorption in the NIR region at the first oxidation stage, due to delocalization from a neutral TPA center to the TPA cation radical center.

The electrochromic switching stability of TPA-based polymers is primarily influenced by the stability of cation radicals. Nelson and Adam et al. reported that electron-donating substituents such as methoxy group tend to stabilize the cation radicals, while electron-withdrawing groups such as nitro group have the opposite effect [27,28]. Two crucial factors affecting the stability of cation radicals are identified. Firstly, the electron-donating or electron-withdrawing effect of substituents contributes to stability. The electron-donating substituents such as methoxy group adjacent to the benzene ring attached to the electroactive nitrogen center stabilizes cation radicals. Since cation radicals are considered to be electron deficient, we would expect an electron-donating group to stabilize cation radicals. Any time a charge of cation radical can be dispersed or delocalized by inductive or resonance effect, a system will be stabilized. Secondly, the resonance effect of electron delocalization over the electroactive nitrogen centers contributes to stability. The TPPA cation radical as a symmetrical delocalized class III, the electron is delocalized over the two redox centers, as shown in Figure 5.

In this study, the polymers **9** with four electroactive TPA cores in the repeating unit were synthesized. Electrochromic stability was compared between **9a** and high electrochromic stability of TPA-based PAs containing the electron-donating methoxy-substituents at the *para*- and/or *ortho*-position of phenyl rings such as **Ref PA1** and **Ref PA2**, as summarized in Table 6. The methoxy group is an electron-donating substituent because of its contribution of electron density to the benzene ring through resonance. The chemical structure of **Ref PA2** contains both *ortho* and *para* methoxy substituents, whereas that of **9a** contains only one *para* methoxy substituent. **PA2** contains more electron-donating methoxy substituents than **9a**; therefore, the electron-donating effect of substituent on the electrochromic stability favors **PA2^+^** over **9a^+^**. **9a** with four electroactive nitrogen centers allows for the formation of more resonance forms for the **9a^+^** cation radical than that of **PA2**. More resonance structures contribute to larger resonance stabilization in a hybrid system described by them. This observation suggests that the resonance effect of the electron delocalized over the electroactive nitrogen centers on the electrochromic stability favoring **9a^+^** over **PA2^+^**. From the result that **9a^+^** is similar to **PA2^+^** in electrochromic stability, it can be seen that the resonance effect and the electron-donating effect almost offset each other. Additionally, **Ref PA1** exhibits lower electrochromic stability than **Ref PA2** or **9a** at the first stage oxidation. The lower electrochromic switching stability is attributed to the fact that **PA1** has the lowest number of electroactive nitrogen centers compared with **PA2** and **9a**. **PA1** possesses two electroactive nitrogen centers and the **PA1^+^** cation radical has a smaller number of resonance structures than **9a^+^** or **PA2^+^**; thus, **PA1^+^** exhibits slightly lower electrochromic stability compared with **9a^+^** and **PA2^+^**. This result suggests that the electron delocalization over all electroactive nitrogen centers significantly stabilize cation radicals. Incorporating multiple redox centers into the polymer chain significantly enhances its electrochromic stability. It is noteworthy that the stability of each cation radical center on the TPA-based polymer is determined by the electron-donating or electron-withdrawing effect of substituents and the resonance effect of electron delocalized over a larger part of the polymer chain.

**Figure 10:**
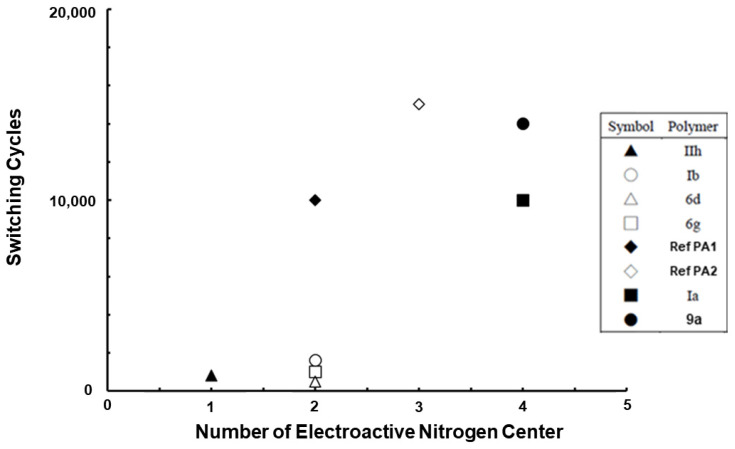
Switching cycles correlated to number of the electroactive nitrogen centers of triarylamine-based polymers with 10% decay of its CE in continuous over 500 cycles at the first oxidation stage. Referenced polymers: **IIh**, Chang C.W. 2007; **Ib**, Chang C.W. 2008; **6d**, Hsiao S.H. 2009; **Ref PA1**, Yen H.J. 2009; **Ref PA2**, Chern Y.T. 2024; **Ia** Liou G.S. 2009; **9a**, this study.

The stability of the polymer for long term switching between oxidized and neutral states is important for practical application [41,42,43]. To confirm the relationships between electrochromic stability and number of the electroactive nitrogen centers, switching cycles correlated to number of the electroactive nitrogen centers of triarylamine-based polymers with 10% decay of its CE in continuous over 500 cycles at the first oxidation stage are presented in Figure 10. As shown in Figure 10, the polymers containing more electroactive nitrogen centers exhibit higher electrochromic stability at the first oxidation stage. For example, their electrochromic stabilities increase as the number of electroactive nitrogen centers increases (**9a**^+^ > **Ref PA1**^+^ > **6g^+^** > **IIh^+^**) at the first stage oxidation; moreover, these polymers contain the electron-donating methoxy substituents adjacent to the benzene ring attached to the electroactive nitrogen center that stabilizes cation radicals. Compared to all other triarylamine-based polymers, **9a** exhibited the highest electrochromic stability. Both of **9a** and **Ia** contain four electroactive nitrogen centers. **9a** exhibits higher electrochromic stability than **Ia**. The reasons for the different stability are currently unclear. However, **Ia** containing four electroactive nitrogen centers also shows high electrochromic stabilities in over 10,000 continuous cycles at the first and second oxidation stages [13].

**Figure 11:**
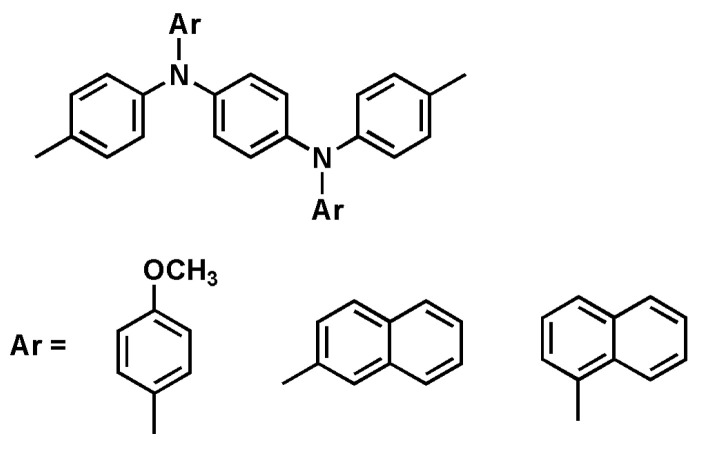
Typical bis(triarylamine) segments used for electrochromic polymers.

Multicolor and high stability of electrochromic materials have attracted considerable attention for their potential applications in displays. The electrochromic switching stability of TPA-based polymers is significantly influenced by different chemical structure. For example, TPA-based polymers containing two electroactive nitrogen centers with different substituents showed significantly different electrochromic stability. Typical chemical structures of bis(triarylamine) segments containing two electroactive nitrogen units are shown in Figure 11 [12,44,45].

TPA-based polymers with *para*-methoxyphenyl substituents exhibited excellent electrochromic stability with their electroactivity decaying less than 10% even after 10,000 switching cycles at the first oxidation stage [12]. However, TPA-based polymers with naphthyl substituents reveal that their electroactivity was retained at 90% after only 100 switching cycles at the first oxidation stage [44,45]. High electrochromic stability of polymers with *para*-methoxyphenyl substituents is attributed to the fact that electron-donating methoxy substituents tend to stabilize the cation radicals. Herein, we focused on specifically designed TPA-based PAs with four electroactive nitrogen centers containing 4-methoxyphenyl or 1-naphthyl groups. Chemical structures of PAs containing four electroactive nitrogen units are indicated in Figure 1.

Interestingly, **9a** and **10d** exhibited approximately high electrochromic stability (only 3.7% and 6.4% decay of its coloration efficiency (CE) at 445 and 446 nm after 14,000 switching cycles, respectively) at the first oxidation stage. **10d** with two naphthyl substituents exhibited unexpected high electrochromic stability. High electrochromic stability of **10d** may be attributed to the resonance effect of four electroactive nitrogen centers and electron-donating effect of two methoxy groups being sufficient to stabilize the cation radical at the first oxidation stage. Importantly, different multicolor of high electrochromic stability of polymers, which allow for multicolor tunability by adjusting Ar_1_ substituents, can be developed from the specifically designed structures. New functional materials can be created from the designed structures. For example, electrofluorochromic materials with high electrochromic stability can be developed by introduction of aryl substituents as fluorescent groups, such as anthracene [46] and pyrene [47].

## 4. Conclusions

Two series of novel aromatic polyamides (**9** and **10**) were successfully prepared from the newly synthesized triarylamine-based diamine monomers **3** and **7** with common aromatic dicarboxylic acids via phosphorylation polyamidation reactions. The polyamides exhibited useful electrochromic characteristics, including multiple color changes, fast response speed, high optical contrast, and excellent electrochromic stability. At the first oxidation stage, the polymers revealed enhanced electrochromic stability (only 3.7 to 6.4% decay in its CE at about 445 nm after 14,000 switching cycles) compared to all other TPA-based polymers to date. The polyamides also showed strong absorption in the near-infrared region upon oxidation. Polymers with multicolored electrochromism and high electrochemical stability can be developed by incorporation of multiple triarylamine cores in the repeat unit and electron-donating methoxy groups on the active sites of the triphenylamine units.

## Figures and Tables

**Table 1:** Optical and electrochemical properties of PAs.

	Solution/Film (nm)		Oxidation Potential (V)							
						*E*_1/2_ ^c^				
Polymer	λ_max_ ^a^	λ_onset_ ^a^	*E*_g_ ^b^ (eV)	*E*_onset_	1st	2nd	3rd	4th	HOMO ^d^ (eV)	LUMO ^e^ (eV)
**9a**	277 (319)	341 (420)	2.95	0.27	0.42	0.61	0.93	1.06	4.78	1.83
**9b**	275 (317)	365 (418)	2.97	0.26	0.42	0.62	0.93	1.05	4.78	1.81
**9c**	323 (314)	407 (428)	2.90	0.26	0.42	0.61	0.94	1.04	4.78	1.88
**10d**	343 (355)	410 (416)	2.98	0.28	0.43	0.67	1.11	-	4.79	1.81
**10e**	333 (333)	427 (452)	2.74	0.23	0.39	0.62	1.08	-	4.75	2.01
**10f**	331 (331)	429 (440)	2.82	0.21	0.38	0.62	1.08	-	4.74	1.92

^a^ UV/vis absorption measurement in NMP (1 × 10^−5^ M) at room temperature, values in parentheses are polymer film state. ^b^ The data were calculated from polymer films by the equation: *E*_g_ = 1240/λ_onset_ (energy gap between HOMO and LUMO). ^c^ From cyclic voltammograms versus Ag/AgCl in CH_3_CN. *E*_1/2_: Average potential of the redox couple peaks. ^d^ The HOMO energy levels were calculated from cyclic voltammetry and were referenced to ferrocene (4.8 eV; *E*_1/2_ = 0.44 V). ^e^ LUMO = HOMO − *E*_g_.

**Table 2:** Optical and electrochemical data collected for coloration efficiency measurements of PA **9a** at 0.4 V.

Cycles ^a^	ΔOD_1409_ ^b^	ΔT(%)_1409_ ^c^	Q_1409_ ^d^ (mC/cm^2^)	η ^e^ (cm^2^/C)	Decay_1409_ ^f^ (%)
1	0.387 (0.463) ^g^	59.0 (65.6)	1.27 (1.70)	305 (272)	0.00 (0.00)
1000	0.387 (0.461)	59.0 (65.4)	1.27 (1.70)	305 (272)	0.00 (0.00)
2000	0.381 (0.455)	58.4 (64.9)	1.26 (1.68)	303 (271)	0.66 (0.37)
3000	0.378 (0.455)	58.1 (64.9)	1.25 (1.68)	302 (271)	0.98 (0.37)
4000	0.373 (0.454)	57.6 (64.8)	1.24 (1.67)	301 (271)	1.31 (0.37)
5000	0.372 (0.451)	57.5 (64.6)	1.24 (1.67)	300 (271)	1.64 (0.37)
6000	0.370 (0.447)	57.3 (64.3)	1.23 (1.65)	300 (270)	1.64 (0.73)
7000	0.366 (0.447)	56.9 (64.3)	1.22 (1.65)	300 (270)	1.64 (0.73)
8000	0.365 (0.445)	56.8 (64.1)	1.22 (1.65)	299 (270)	1.97 (0.73)
9000	0.356 (0.439)	55.9 (63.6)	1.20 (1.63)	298 (269)	2.29 (1.10)
10,000	0.348 (0.438)	55.1 (63.5)	1.18 (1.63)	295 (269)	3.28 (1.10)
11,000	0.343 (0.437)	54.6 (63.4)	1.17 (1.63)	294 (269)	3.61 (1.10)
12,000	0.342 (0.433)	54.5 (63.1)	1.17 (1.62)	293 (267)	3.93 (1.84)
13,000	0.340 (0.426)	54.3 (62.5)	1.16 (1.61)	293 (264)	3.93 (2.94)
14,000	0.337 (0.419)	54.0 (61.9)	1.15 (1.60)	292 (262)	4.26 (3.68)

^a^ Times of cyclic scan by applying potential step; 0.0 V ↹ 0.4 V (V vs. Ag/AgCl). ^b^ Optical density change at 1409 nm. ^c^ Optical transmittance change at 1409 nm. ^d^ Ejected charge, determined from in situ experiments. ^e^ Coloration efficiency is derived from the equation η = ∆OD/Q. ^f^ Decay of coloration efficiency after cyclic scans. ^g^ Data in parentheses are optical density change at 445 nm.

**Table 3:** Optical and electrochemical data collected for coloration efficiency measurements of PA **9a** at 0.6 V.

Cycles ^a^	ΔOD_1409_ ^b^	ΔT (%) ^c^	Q (mC/cm^2^) ^d^	η (cm^2^/C) ^e^	Decay (%) ^f^
1	0.912	87.8	3.43	266	0.00
1000	0.898	87.4	3.40	264	0.75
2000	0.898	87.4	3.40	264	0.75
3000	0.884	86.9	3.36	263	1.13
4000	0.854	86.0	3.32	257	3.38
5000	0.837	85.4	3.29	255	4.13
6000	0.828	85.1	3.26	254	4.51
7000	0.823	85.0	3.24	254	4.51
8000	0.769	83.0	3.17	243	8.65

^a^ Times of cyclic scan by applying potential step: 0.0 V ↹ 0.6 V (V vs. Ag/AgCl). ^b^ Optical density change at 1409 nm. ^c^ Optical transmittance change at 1409 nm. ^d^ Ejected charge, determined from in situ experiments. ^e^ Coloration efficiency is derived from the equation η = ∆OD/Q. ^f^ Decay of coloration efficiency after cyclic scans.

**Table 4:** Optical and electrochemical data collected for coloration efficiency measurements of PA **10d** at 0.5 V.

Cycles ^a^	ΔOD ^b^	ΔT (%) ^c^	Q ^d^ (mC/cm^2^)	η ^e^ (cm^2^/C)	Decay ^f^ (%)
1	0.653 (0.439) ^g^	77.8 (63.6)	1.71 (1.86)	381 (236)	0.00 (0.00)
1400	0.647 (0.432)	77.5 (63.0)	1.71 (1.85)	379 (234)	0.52 (0.85)
2800	0.638 (0.430)	77.0 (62.8)	1.69 (1.84)	377 (234)	1.05 (0.85)
4200	0.629 (0.427)	76.5 (62.6)	1.68 (1.83)	375 (233)	1.57 (1.27)
4600	0.617 (0.421)	75.8 (62.1)	1.66 (1.82)	372 (231)	2.36 (2.12)
7000	0.594 (0.417)	74.5 (61.7)	1.62 (1.82)	368 (230)	3.41 (2.54)
8400	0.558 (0.415)	72.3 (61.5)	1.55 (1.81)	359 (229)	5.77 (2.97)
9800	0.547 (0.407)	71.6 (60.8)	1.54 (1.80)	356 (227)	6.56 (3.81)
11,200	0.527 (0.401)	70.3 (60.2)	1.51 (1.78)	349 (225)	8.40 (4.66)
12,600	0.522 (0.393)	69.9 (59.5)	1.51 (1.76)	347 (224)	8.92 (5.08)
14,000	0.501 (0.383)	68.4 (58.6)	1.46 (1.73)	343 (221)	9.97 (6.35)

^a^ Times of cyclic scan by applying potential step: 0.0 V ↹ 0.5 V (V vs. Ag/AgCl). ^b^ Optical density change at 1346 nm. ^c^ Optical transmittance change at 1346 nm. ^d^ Ejected charge, determined from in situ experiments. ^e^ Coloration efficiency is derived from the equation η = ΔOD/Q. ^f^ Decay of coloration efficiency after cyclic scans. ^g^ Data in parentheses are optical density change at 446 nm.

**Table 5:** NIR absorption of polymers at the first oxidation stage.

Polymer Code	Potential ^a^	λ_max_ ^b^	Absorption Range ^b^
	(V)	(nm)	(nm)
**9a**	0.40	1409	800–1800
**Ref PA1**	0.75	1062	800–1500
**Ref PA2**	0.40	1252	800–1700

^a^ Applied potential for the first stage oxidation. ^b^ NIR absorption.

**Table 6:** Long-term electrochromic stability of polymers.

Polymer	Potential (V)	CE (cm^2^/C) ^a^	Cycles ^b^	ΔT (%) ^c^	Decay (%) ^d^	Ref.
**9a**	0.0 ↔ 0.40	272	14,000	65.6	3.7	This study
**Ref PA1**	0.0 ↔ 0.70	388	10,000	54.0	4.9	[12]
**Ref PA2**	0.0 ↔ 0.40	195	15,000	54.0	4.1	[32]

^a^ Initial coloration efficiency. ^b^ Times of cyclic scan by applying potential step. ^c^ Initial contrast of optical transmittance change at 445 nm for **9a**, 433 nm for **Ref PA1**, 422 nm for **Ref PA2**. ^d^ Decay of coloration efficiency after cyclic scans.

## Data Availability

Data are contained within the article and Supplementary Materials.

## Supplementary Materials

The following supporting information can be downloaded at: https://www.mdpi.com/article/10.3390/polym16121644/s1, Figure S1: FT-IR spectra of compounds **2**, **3**, and PA **9a**. Figure S2: FT-IR spectra of compounds **5**–**7** and PA **10e**. Figure S3: (a) ^1^H and (b) ^13^C NMR spectra of dinitro compound **2** in CDCl_3_. Figure S4: (a) H-H COSY and (b) C-H HMQC NMR spectra of dinitro compound **2** in CDCl_3_. Figure S5: (a) ^13^C and (b) C-H HMQC NMR spectra of diamine monomer **3** in pyridine-*d*_5_. Figure S6: (a) ^1^H, (b) ^13^C, and (c) C-H HMQC NMR spectra of compound **5** in DMSO-*d*_6_. Figure S7: (a) ^1^H and (b) ^13^C NMR spectra of compound **6** in DMSO-*d*_6_. Figure S8: (a) H-H COSY and (b) C-H HMQC NMR spectra of compound **6** in DMSO-*d*_6_. Figure S9: (a) H-H COSY and (b) C-H HMQC NMR spectra of diamine monomer **7** in DMSO-*d*_6_. Figure S10: ^1^H NMR spectrum of PA **9c** in DMSO-*d*_6_. Figure S11: ^1^H NMR spectrum of PA **10d** in DMSO-*d*_6_. Figure S12: TGA and TMA thermograms of PA **10d** at a heating rate of 10 °C/min and 20 °C/min, respectively. Figure S13: Potential step absorptometry and current consumption of PA **10d** (in CH_3_CN with 0.1 M TBAP as the supporting electrolyte) by applying a potential step 0.0 V ↹ 0.7 V, and cycle time of 20 s for color efficiency from 281 cm^2^/C (1st cycle) to 250 cm^2^/C (6000th cycle). Figure S14: Potential step absorptometry during the continuous cycling test of PA **10d** (in CH_3_CN with 0.1 M TBAP as the supporting electrolyte) by switching potentials step 0.0 V ↹ 0.5 V with a cycle time of 5 h and 5 min for coloring and bleaching processes, respectively. Figure S15: Absorbance profile of **Ref PA1**, **PA2**, and PA **9a** thin-film on ITO-glass electrode in 0.1 M TBAP/CH_3_CN at different applied potentials. Table S1: Inherent viscosity, molecular weights, and solubility of PAs. Table S2: Thermal properties of PAs. Table S3: Optical and electrochemical data collected for coloration efficiency measurements of PA **10d** at 1268 nm at 0.7 V.
