# Supplementary material for: Redox-Stable and Multicolor Electrochromic Polyamides with Four Triarylamine Cores in the Repeating Unit
Source: Polymers (Basel). 2024 Jun 11;16(12):1644. doi: 10.3390/polym16121644 (PMC11207867; doi:10.3390/polym16121644)
Supplement: Supplementary file 1 [file polymers-16-01644-s001.zip › polymers-3024970-supplementary.pdf]

Supplementary Materials

Redox-Stable and Multicolor Electrochromic Polyamides with Four Triarylamine Cores in the Repeating Unit

Yaw-Terng Chern [1] *, Chien-Cheng Yen [1], Jia-Mao Wang [1], I-Shan Lu [1], Bo-Wei Huang [2], Sheng-Huei Hsiao [2] *

[1] Department of Chemical Engineering, National Taiwan University of Science and Technology, Taipei, Taiwan
[2] Department of Chemical Engineering and Biotechnology, National Taipei University of Technology, Taipei, Taiwan
* Corresponding authors: ytchern@mail.ntust.edu.tw (Y.-T. Chern); shhsiao@ntut.edu.tw (S.-H. Hsiao)

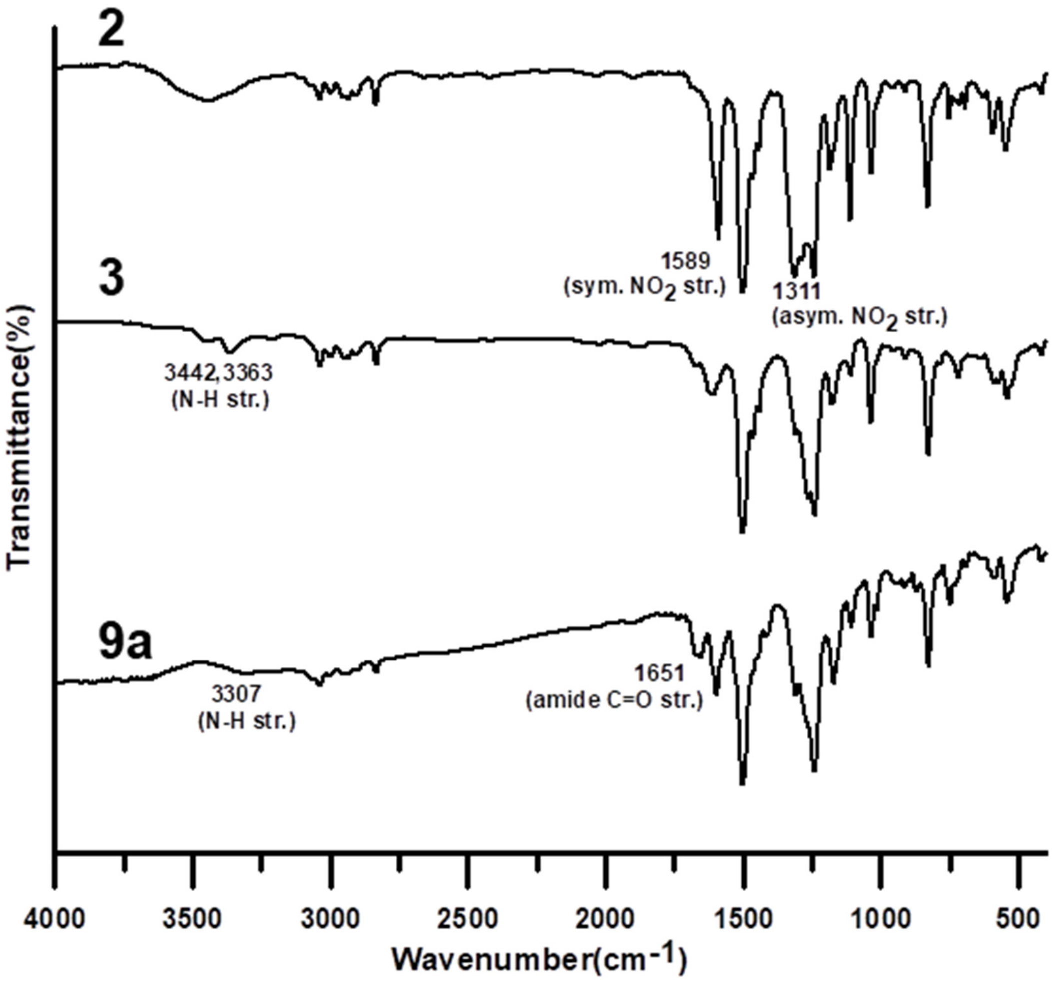

Figure S1. FT-IR spectra of compounds **2**, **3**, and PA **9a**.

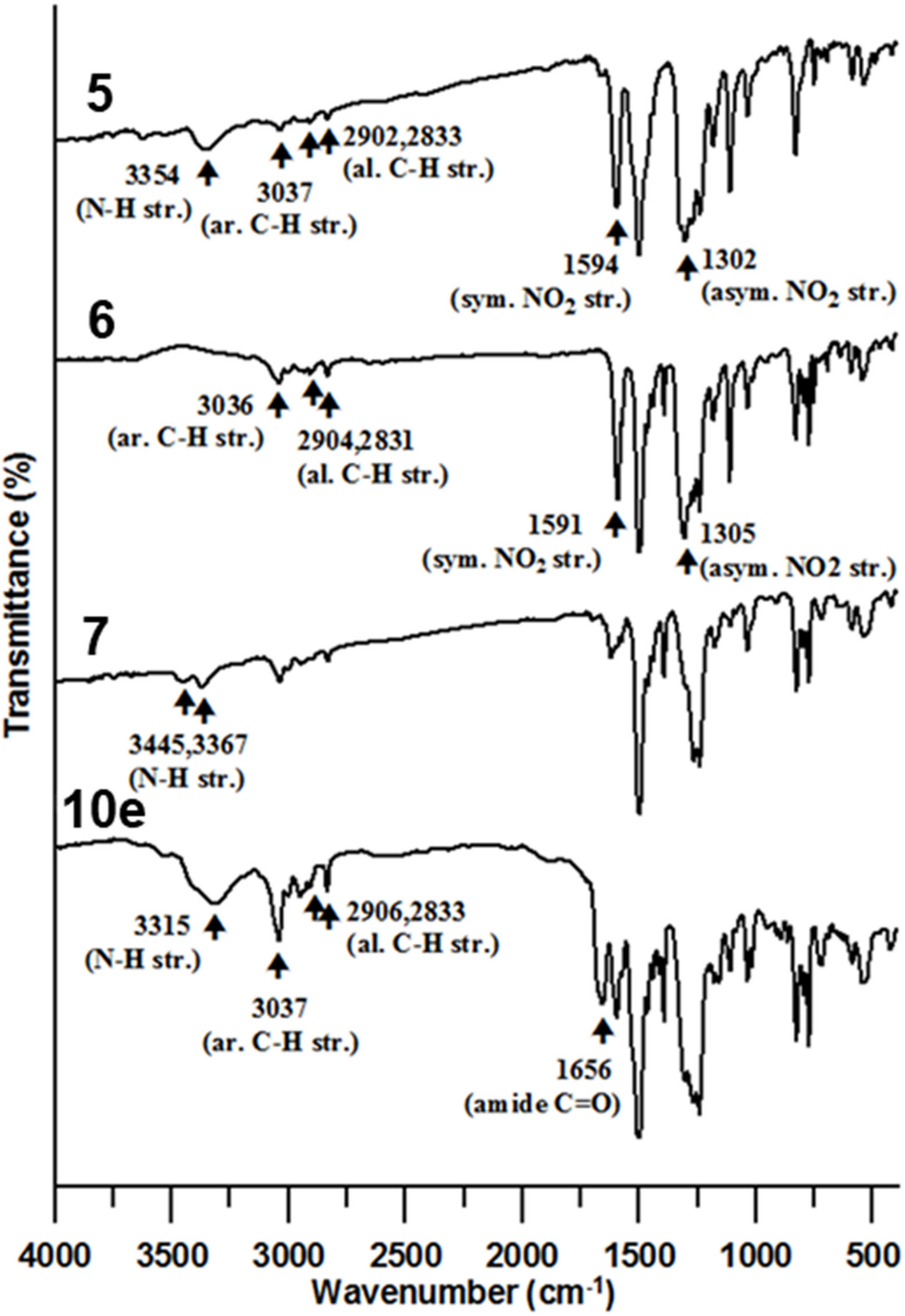

Figure S2. FT-IR spectra of compounds **5-7** and PA **10e**.

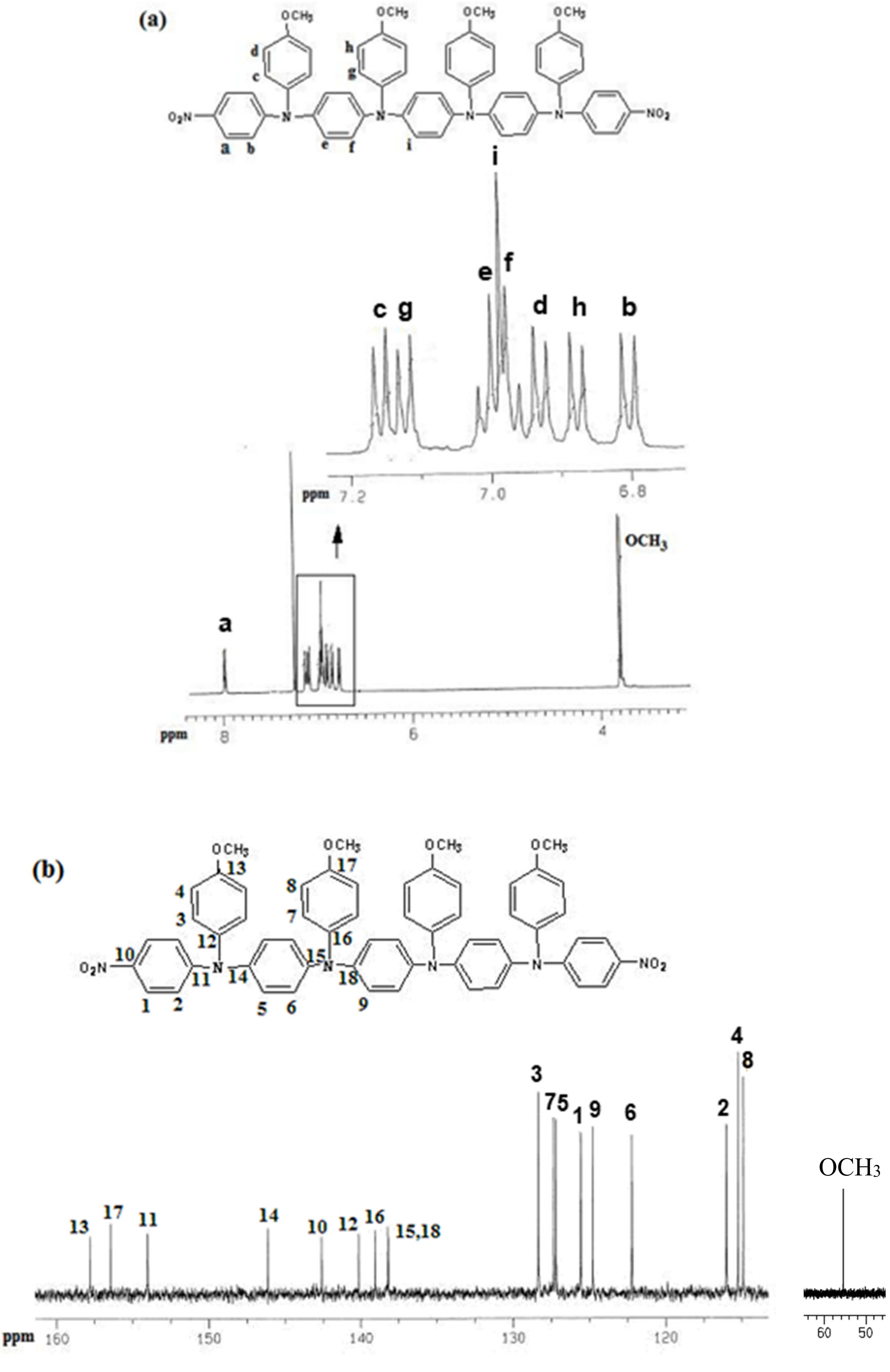

Figure S3. (a) ¹H and (b) ¹³C NMR spectra of dinitro compound **2** in $CDCl_3$.

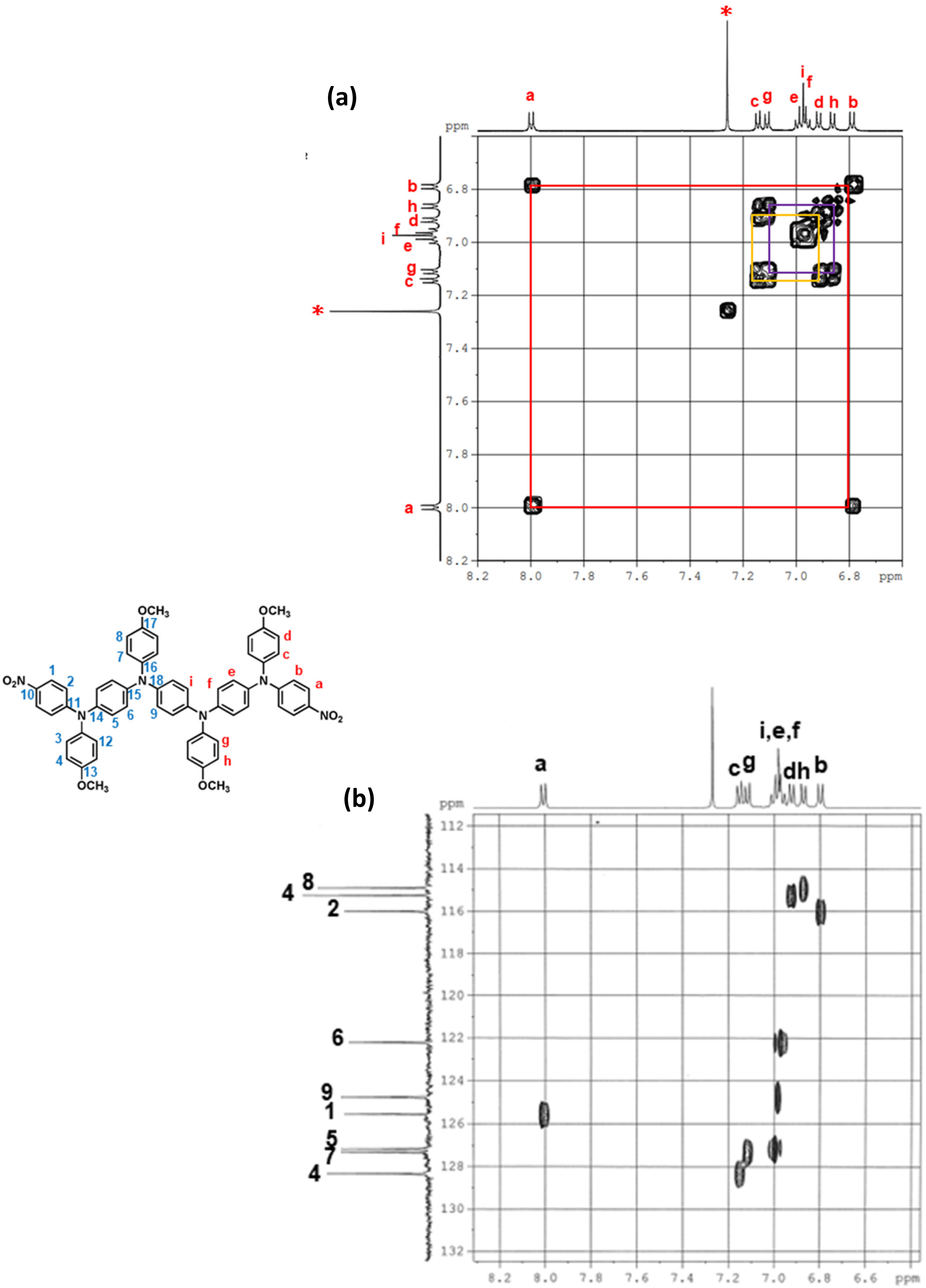

Figure S4. (a) H-H COSY and (b) C-H HMQC NMR spectra of dinitro compound **2** in $CDCl_3$ (* solvent peak).

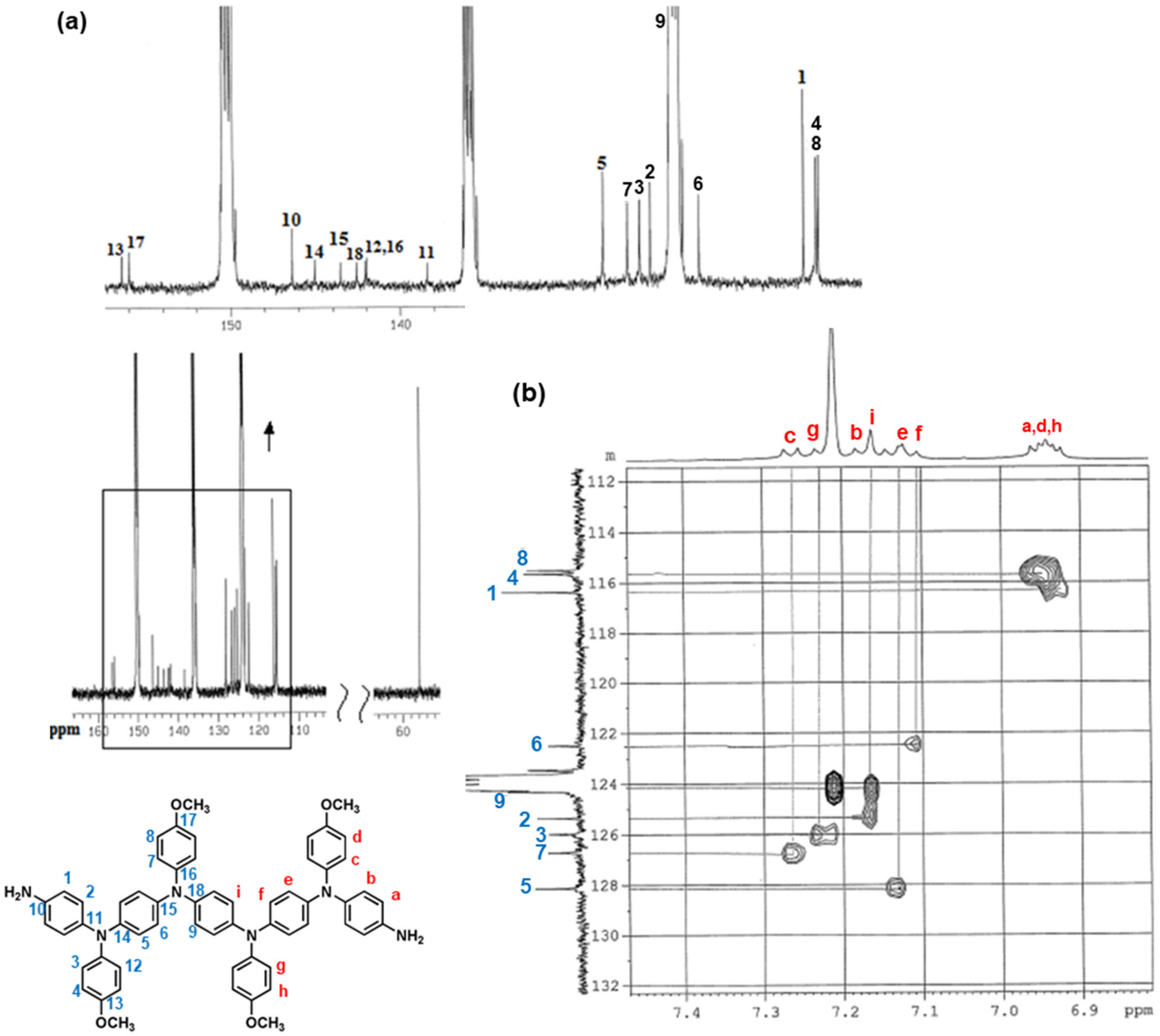

Figure S5. (a) ^{13}C and (b) C-H HMQC NMR spectra of diamine monomer **3** in pyridine-*d*5.

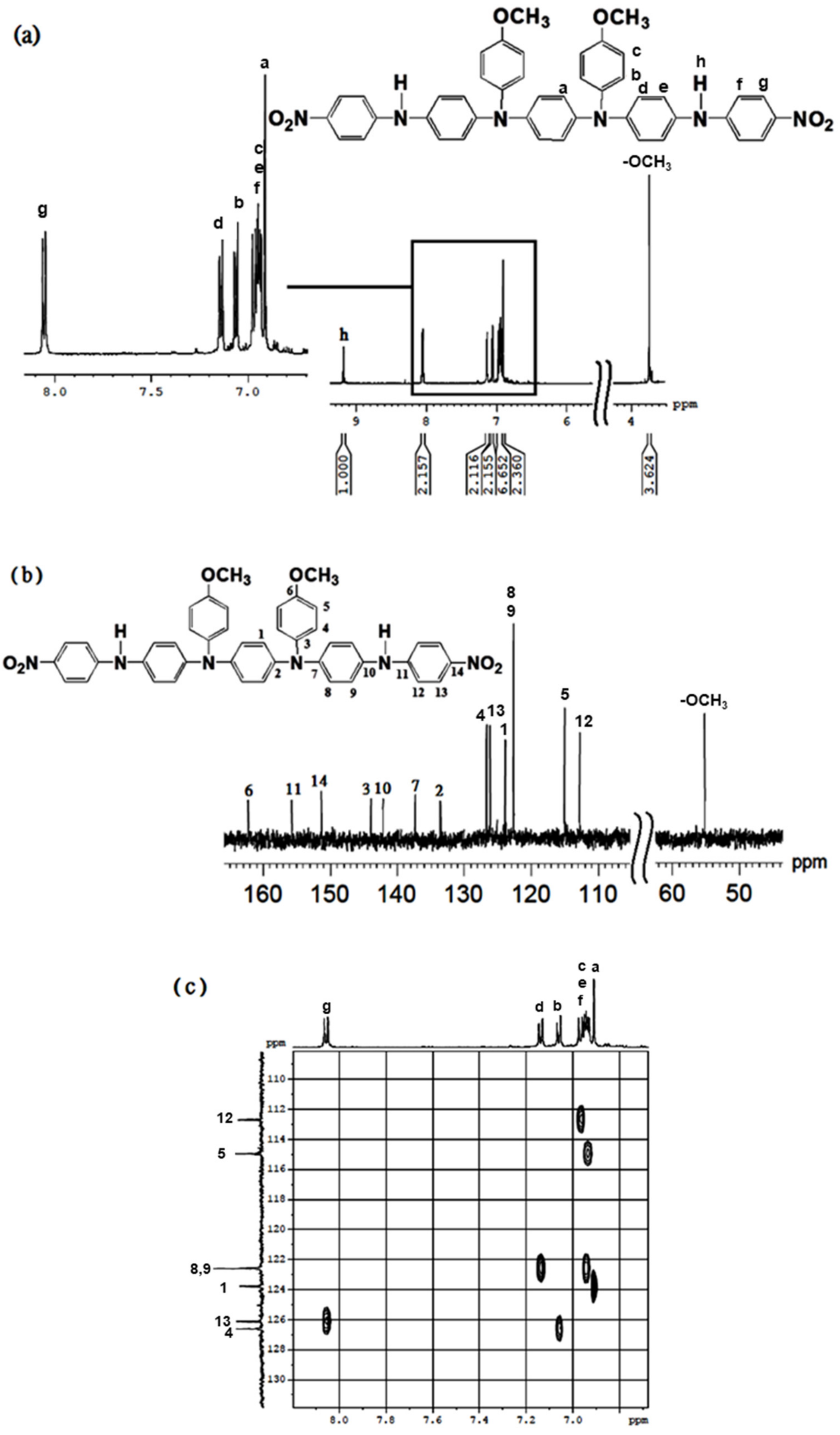

Figure S6. (a) ^{1}H, (b) ^{13}C, and (c) C-H HMQC NMR spectra of compound **5** in DMSO-d_6.

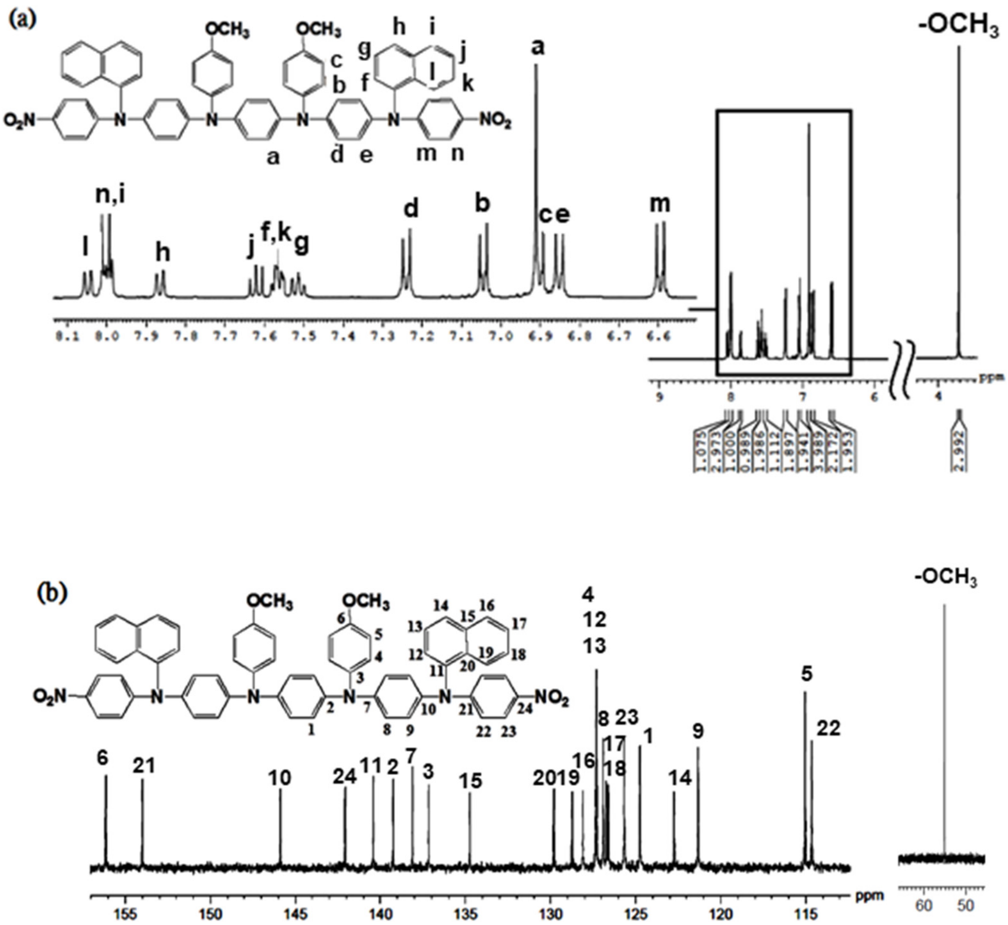

Figure S7. (a) ^1H and (b) ^{13}C NMR spectra of compound **6** in DMSO-d_6.

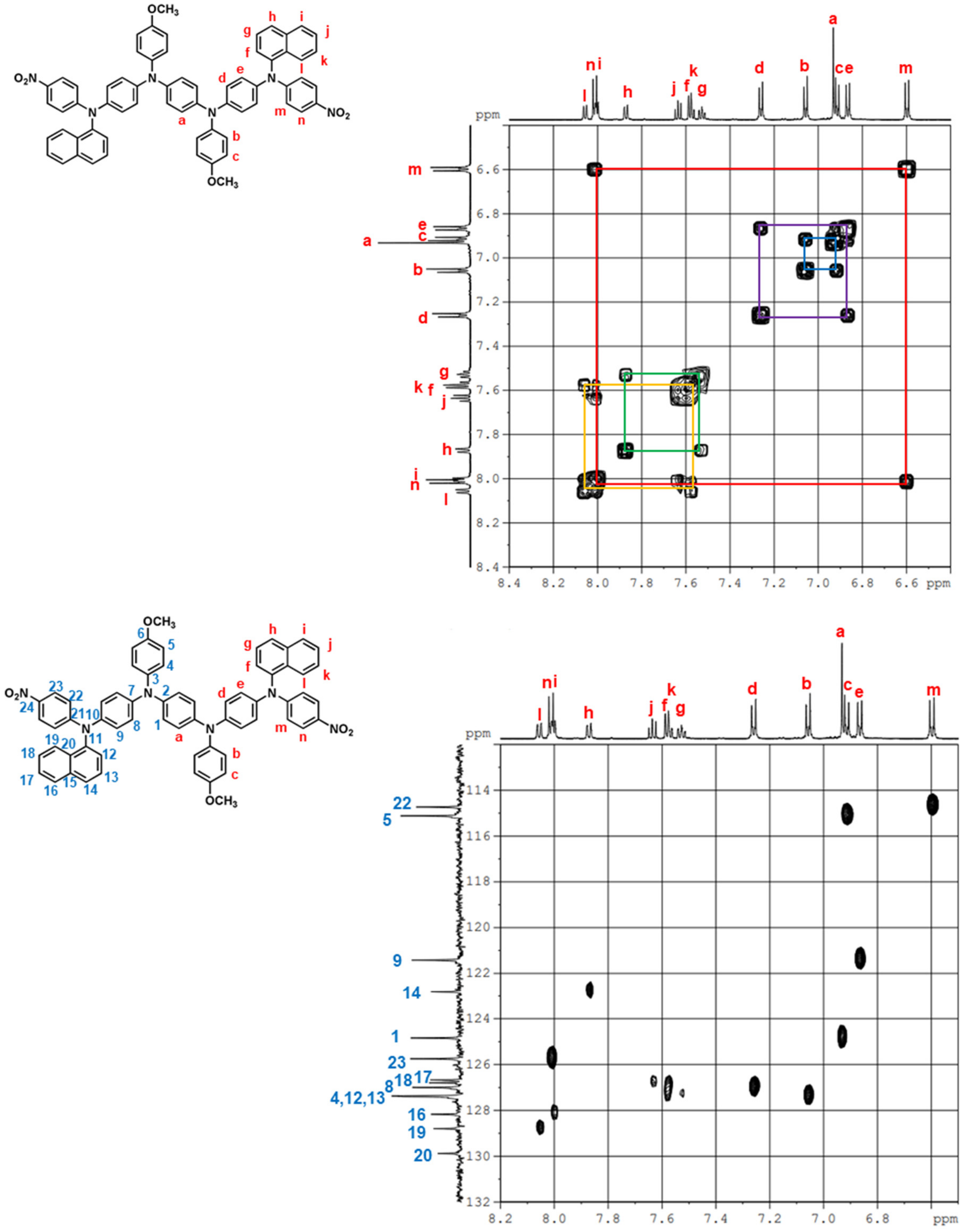

Figure S8. (a) H-H COSY and (b) C-H HMQC NMR spectra of compound **6** in DMSO-d_6.

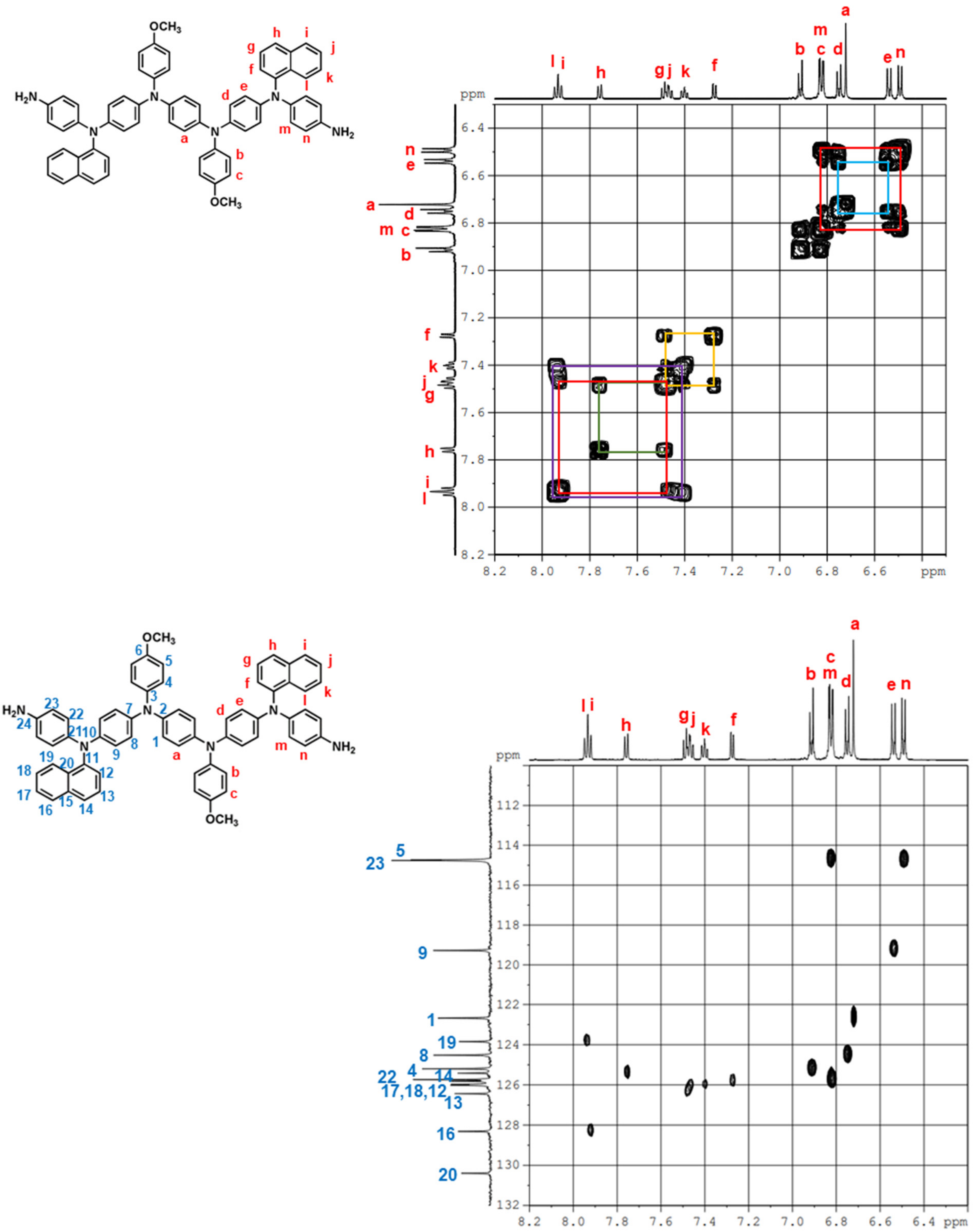

Figure S9. (a) H-H COSY and (b) C-H HMQC NMR spectra of diamine monomer **7** in DMSO-d_6.

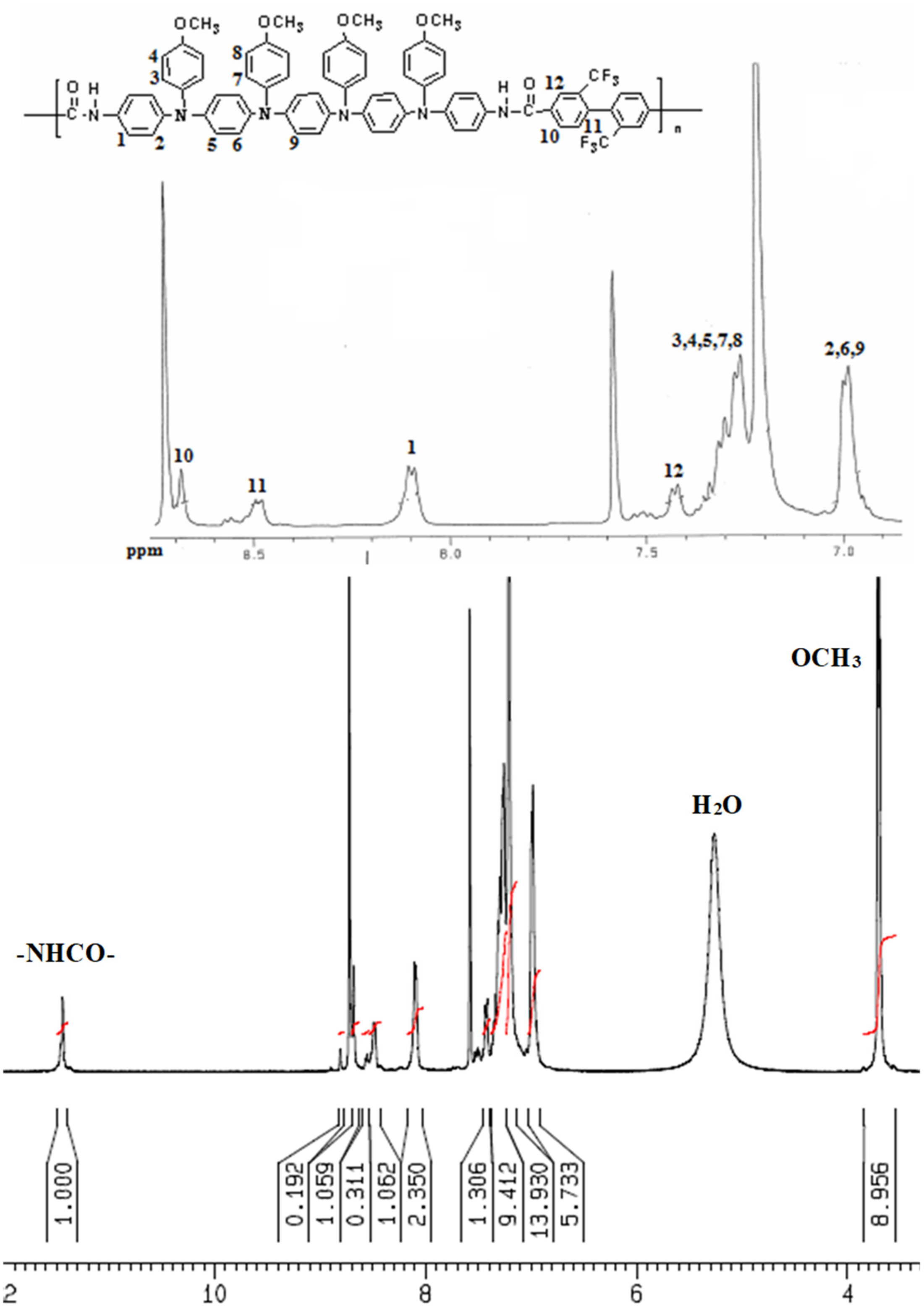

Figure S10. ^{1}H NMR spectrum of PA **9c** in DMSO-d_6.

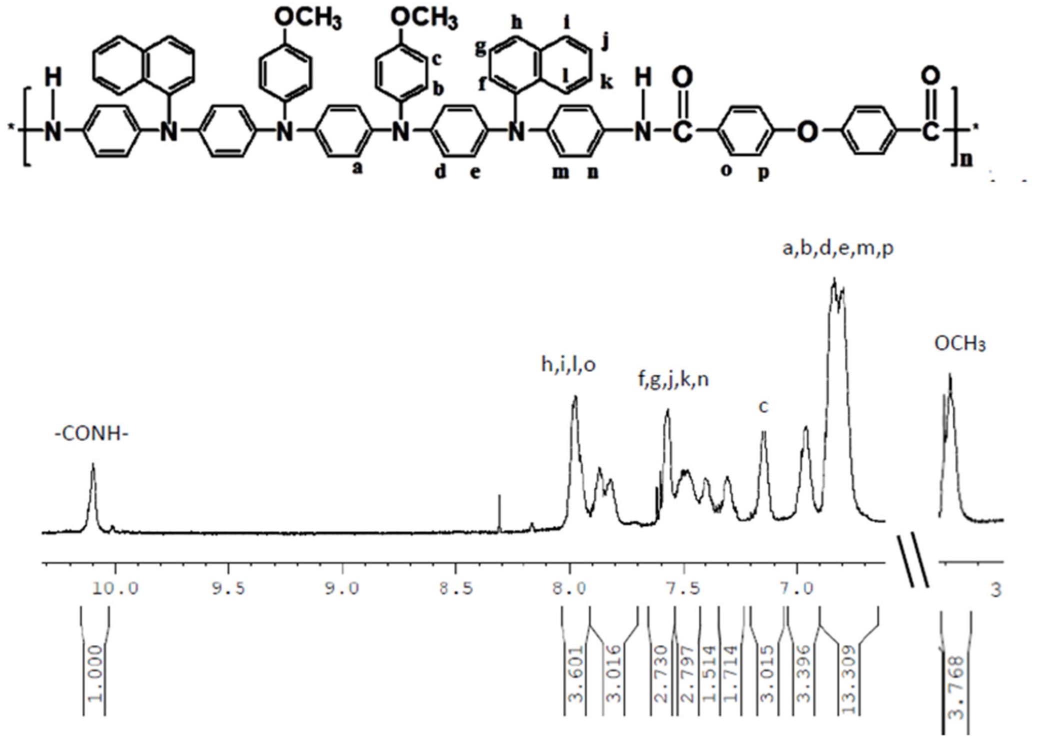

Figure S11. ^{1}H NMR spectrum of PA **10d** in DMSO-d_6.

Table S1. Inherent viscosity, molecular weights, and solubility[a] of PAs

Polymer code[b]	η_{inh} [c] (dL/g)	M_w [d] (kDa)	M_n [d] (kDa)	PDI[e]	NMP	DMAc	*o*-Chloro-phenol	*m*-Cresol
**9a**	0.65	80.4	46.2	1.74	+	+	+	+
**9b**	0.35	43.9	24.8	1.77	+	+	+	++
**9c**	0.36	43.6	24.5	1.78	+	+	+−	+
**10d**	0.47	67.8	38.1	1.78	++	++	++	++
**10e**	0.46	65.8	37.6	1.75	++	++	++	++
**10f**	0.50	67.9	38.6	1.76	++	++	++	++

[a] Qualitative solubility was determined using 0.01 g of polymer in 1 mL of solvent. ++ (soluble at room temperature); + (soluble on heating at 60°C); +− (partially soluble on heating at 60°C). [b] All the polyamides were prepared by a typical synthetic procedure using a similar reaction condition of **10d** as shown in section 2.6. [c] Measured in NMP on 0.5 g/dL at 30°C. [d] Weight-average molecular weights (M_w) and number-average molecular weights (M_n) were obtained via gel permeation chromatography (GPC) using polystyrene as standards and NMP containing 20 mM LiCl as the eluent at a flow rate of 0.30 mL/min at 60°C. [e] Polydispersity index = M_w/M_n.

Table S2. Thermal properties of PAs

Polymer code	T_g (°C)[a]	T_s (°C)[b]	T_d (°C)[c]		Char yield[d] (%)
			In air	In N_2	
**a**	223	218	465	452	65
**b**	204	195	487	446	64
**9c**	208	201	432	421	62
**10d**	256	232	515	502	63
**10e**	261	233	507	502	68
**10f**	268	238	520	510	67

[a] Temperature at which the middle of change of the heat capacity occurred from the second DSC heating scan at a heating rate of 25°C/min. [b] Softening temperature defined as the onset temperature of the probe displacement on the penetration TMA trace. [c] Temperature at which 10% weight loss recorded by thermogravimetry at a heating rate of 20°C/min. [d] Residual wt% at 800°C in nitrogen.

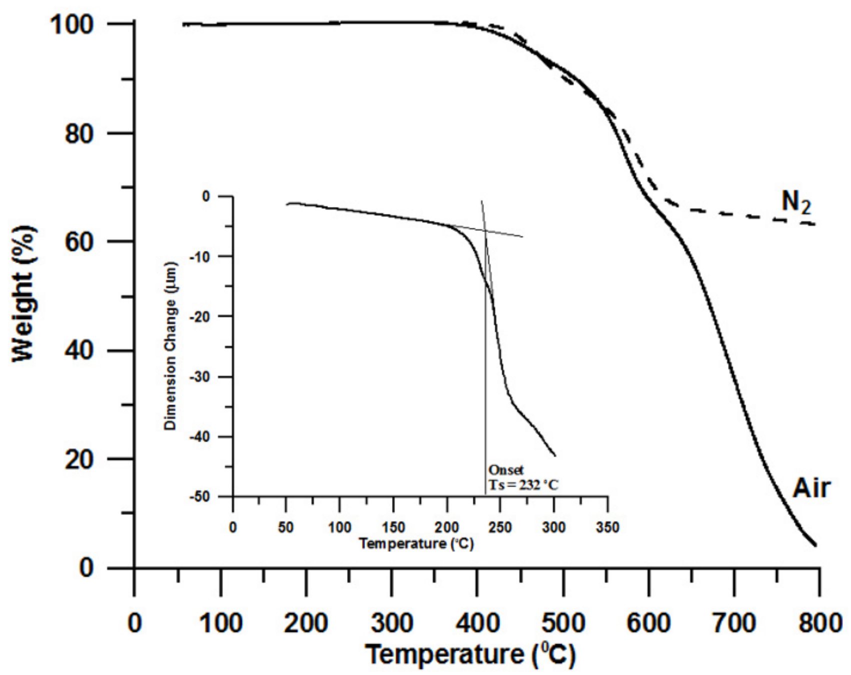

Figure S12. TGA and TMA thermograms of PA **10d** at a heating rate of 10°C/min and 20 °C/min, respectively.

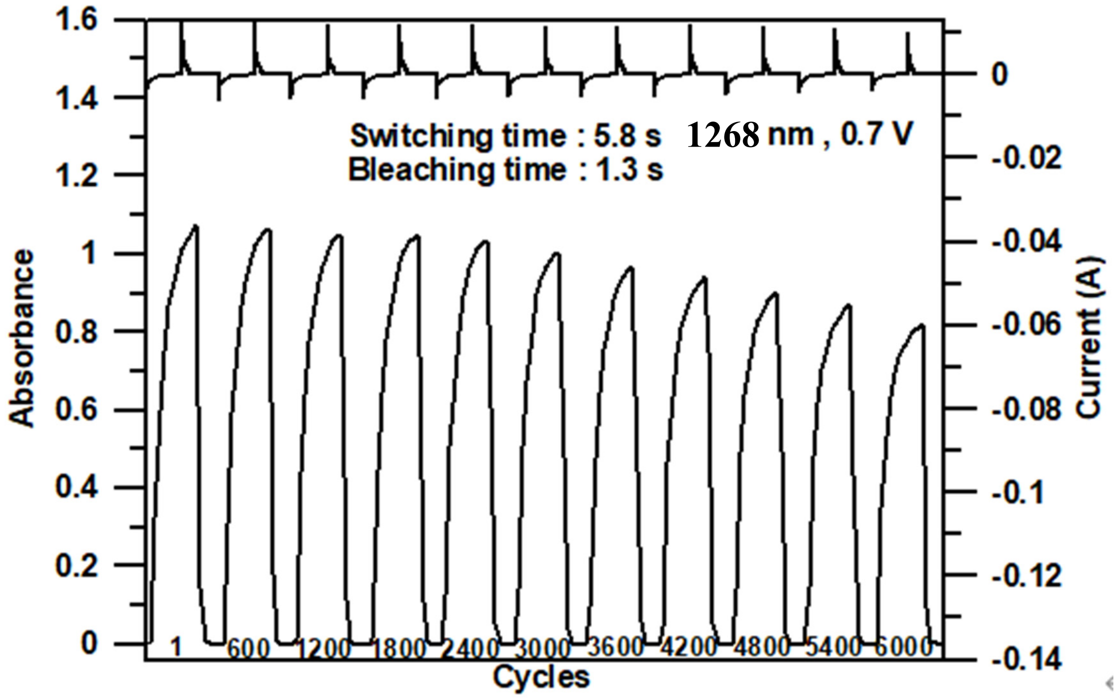

Figure S13. Potential step absorptometry and current consumption of PA **10d** (in CH_3CN with 0.1M TBAP as the supporting electrolyte) by applying a potential step 0.0 V ⇆ 0.7 V, and cycle time of 20s for color efficiency from 281 cm^2/C (1st cycle) to 250 cm^2/C (6000th cycle).

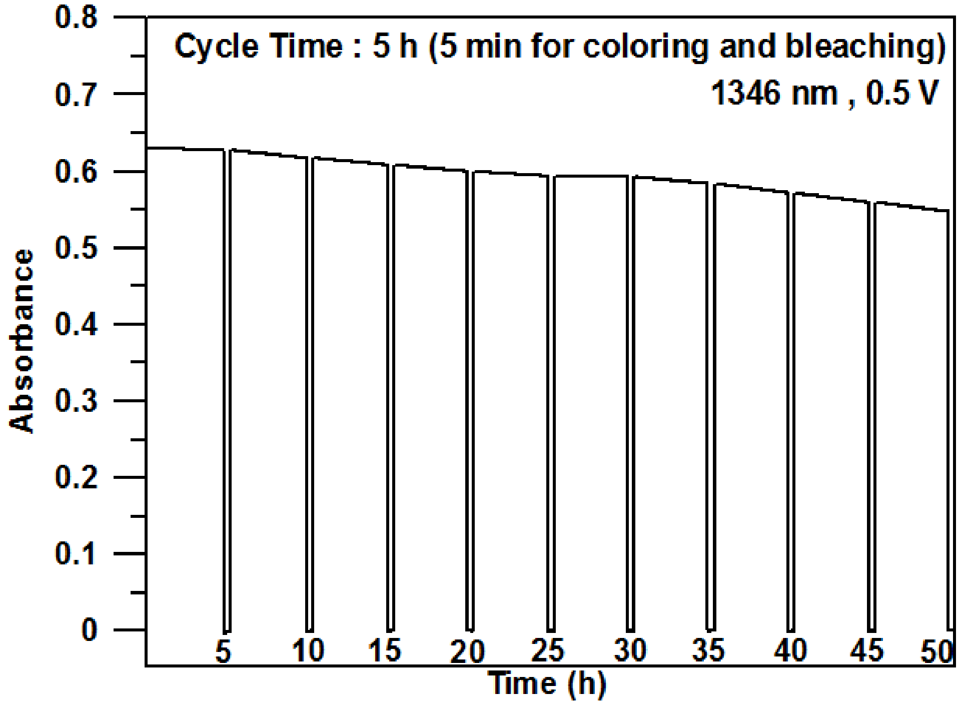

Figure S14. Potential step absorptometry during the continuous cycling test of PA **10d** (in CH_3CN with 0.1M TBAP as the supporting electrolyte) by switching potentials step 0.0 V ⇆ 0.5 V with a cycle time of 5 h and 5 min for coloring and bleaching processes, respectively.

Table S3. Optical and electrochemical data collected for coloration efficiency measurements of PA **10d** at 1268 nm at 0.7 V

Cycles[a]	△OD[b]	△T[c] (%)	Q[d] (mC/cm^2)	η[e] (cm^2/C)	Decay[f] (%)
1	1.074	91.6	3.82	281	0.00
600	1.066	91.4	3.80	280	0.36
1200	1.051	91.1	3.79	278	1.07
1800	1.050	91.1	3.79	277	1.42
2400	1.035	90.8	3.76	276	1.78
3000	1.005	90.1	3.67	274	2.49
3600	0.968	89.2	3.64	266	5.34
4200	0.942	88.6	3.57	264	6.05
4800	0.905	87.6	3.49	259	7.83
5400	0.870	86.5	3.41	255	9.25
6000	0.821	84.9	3.28	250	11.03

[a] Times of cyclic scan by applying potential step: 0.0V→0.7V (V vs. Ag/AgCl). [b] Optical density change at 1268 nm for (7d). [c] Optical transmittance change at 1268 nm for (7d). [d] Ejected charge, determined from in situ experiments. [e] Coloration efficiency is derived from the equation: η = △OD/Q. [f] Decay of coloration efficiency after cyclic scans.

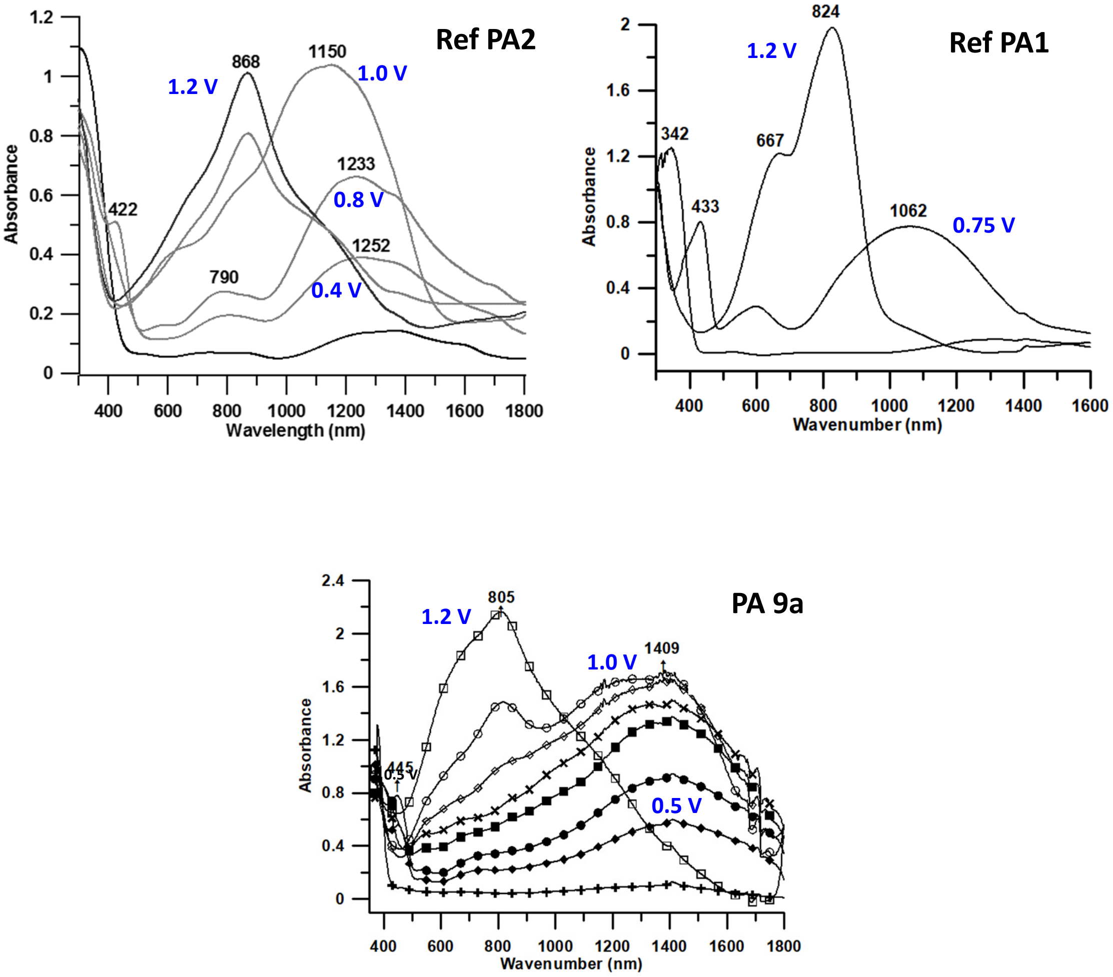

Figure S15. Absorbance profile of **Ref PA1, PA2,** and PA **9a** thin-film on ITO-glass electrode in 0.1 M TBAP/CH_3CN at different applied potentials.
